# Global genetic analyses reveal strong inter-ethnic variability in the loss of activity of the organic cation transporter OCT1

**DOI:** 10.1186/s13073-015-0172-0

**Published:** 2015-06-18

**Authors:** Tina Seitz, Robert Stalmann, Nawar Dalila, Jiayin Chen, Sherin Pojar, Joao N. Dos Santos Pereira, Ralph Krätzner, Jürgen Brockmöller, Mladen V. Tzvetkov

**Affiliations:** Institute of Clinical Pharmacology, University Medical Center Göttingen, Robert-Koch-Str. 40, DE-37075 Göttingen, Germany; Department of Pediatrics and Adolescent Medicine, University Medical Center Göttingen, Robert-Koch-Str. 40, DE-37075 Göttingen, Germany

## Abstract

**Background:**

The organic cation transporter OCT1 (SLC22A1) mediates the uptake of vitamin B1, cationic drugs, and xenobiotics into hepatocytes. Nine percent of Caucasians lack or have very low OCT1 activity due to loss-of-function polymorphisms in *OCT1* gene. Here we analyzed the global genetic variability in *OCT1* to estimate the therapeutic relevance of *OCT1* polymorphisms in populations beyond Caucasians and to identify evolutionary patterns of the common loss of OCT1 activity in humans.

**Methods:**

We applied massively parallel sequencing to screen for coding polymorphisms in 1,079 unrelated individuals from 53 populations worldwide. The obtained data was combined with the existing 1000 Genomes data comprising an additional 1,092 individuals from 14 populations. The identified OCT1 variants were characterized *in vitro* regarding their cellular localization and their ability to transport 10 known OCT1 substrates. Both the population genetics data and transport data were used in tandem to generate a world map of loss of OCT1 activity.

**Results:**

We identified 16 amino acid substitutions potentially causing loss of OCT1 function and analyzed them together with five amino acid substitutions that were not expected to affect OCT1 function. The variants constituted 16 major alleles and 14 sub-alleles. Six major alleles showed improper subcellular localization leading to substrate-wide loss in activity. Five major alleles showed correct subcellular localization, but substrate-specific loss of activity. Striking differences were observed in the frequency of loss of OCT1 activity worldwide. While most East Asian and Oceanian individuals had completely functional OCT1, 80 % of native South American Indians lacked functional *OCT1* alleles. In East Asia and Oceania the average nucleotide diversity of the loss-of-function variants was much lower than that of the variants that do not affect OCT1 function (ratio of 0.03) and was significantly lower than the theoretically expected heterozygosity (Tajima’s D = −1.64, *P* < 0.01).

**Conclusions:**

Comprehensive genetic analyses showed strong global variations in the frequency of loss of OCT1 activity with selection pressure for maintaining OCT1 activity in East Asia and Oceania. These results not only enable pharmacogenetically-based optimization of drug treatment worldwide, but may help elucidate the functional role of human OCT1.

**Electronic supplementary material:**

The online version of this article (doi:10.1186/s13073-015-0172-0) contains supplementary material, which is available to authorized users.

## Background

The polyspecific organic cation transporter OCT1 (SLC22A1) is strongly expressed in human hepatocytes and facilitates uptake of organic cations from the sinusoidal blood. Known substrates of OCT1 are vitamin B1, toxins like 1-methyl-4-phenylpyridinium (MPP^+^) and monocrotaline, and clinically relevant drugs like metformin, morphine, tropisetron, tramadol, and others [1–9].

The human *OCT1* gene is highly polymorphic. Forty percent of Caucasians carry one and a further 9 % carry two of the following loss-of-function polymorphisms: arginine_61_-to-cysteine (Arg61Cys), cysteine_88_-to-arginine (Cys88Arg), glycine_401_-to-serine (Gly401Ser), glycine_465_-to-arginine (Gly465Arg), or a deletion of methinone_420_ (Met420del) [4, 10, 11]. Depending on the substrate and zygosity, these individuals completely lack or have substantially reduced OCT1 activity [2–5]. Significant changes in pharmacokinetics and, as a consequence, in clinical effects of several drugs were observed in the carriers of loss-of-function *OCT1* polymorphisms [3–5, 12].

The reasons for the frequent loss of OCT1 activity remain elusive. Although a number of endogenous molecules, and recently vitamin B1, have been found to be substrates of OCT1 [1, 13], no major differences in the vitality or fertility were observed between the wild-type and *Oct1* knockout mice [14]. However, the liver concentrations of the model substrate tetraethylammonium and the drug metformin were significantly reduced in *Oct1* knockout mice [14, 15], suggesting an important role of OCT1 in the hepatic uptake of drugs and xenobiotics with cationic or weak basic structure. Recently, reduced hepatic uptake of vitamin B1 in Oct1 knockout mice was reported to lead to a decreased risk of hepatic steatosis [1]. This decreased risk may improve fitness and provide a survival advantage, although long-term studies in mice and studies in humans to support this are still missing.

Several studies analyzed the genetic variability in OCT1 in detail. Kerb *et al.* resequenced *OCT1* coding and promoter regions in 57 Caucasians and analyzed the polymorphisms they identified in another 190 unrelated Caucasians [10]. They found eight non-synonymous polymorphisms, three of which affected OCT1 activity. Leabman *et al.* screened for coding genetic variants in *OCT1* and 23 other membrane transporters in 247 unrelated individuals: 100 European Americans, 100 African Americans, 30 Asians, 10 Mexicans, and seven Pacific Islanders [16]. Five out of the 15 non-synonymous variants identified by Leabman *et al.* were shown to strongly decrease OCT1 activity, and three of them completely abolished activity [11]. With a non-synonymous nucleotide diversity of 5.11 × 10^−4^ and a ratio of non-synonymous to synonymous nucleotide diversity of 0.46, OCT1 had the strongest genetic variability among the organic cation transporters of the SLC22 family [16]. Several studies analyzed the genetic variability of OCT1 in Asians. A total of 230 Japanese individuals were resequenced [17–19] and the identified non-synonymous variants were functionally characterized [17, 20, 21]. The number and the frequency of polymorphisms causing loss of or decreased OCT1 function was much lower in Asians than in Caucasians [17], suggesting the presence of substantial inter-ethnic variability in genetically determined loss of OCT1 activity.

In this study we performed a global-scale population genetics analysis and detailed functional analyses in order to generate a world map of genetically determined loss of OCT1 activity. This map should help to elucidate the potential role of *OCT1* polymorphisms in interethnic differences in drug therapy. Furthermore, we were looking for global patterns that may point to factors causing selection pressure for the frequent loss of OCT1 activity. This study expanded the number of individuals and world regions analyzed far beyond the scope of previously published analyses of genetic variability in OCT1. Here we analyzed data from 2,171 unrelated individuals from 67 worldwide populations. Using massively parallel sequencing we screened the OCT1 gene for amino acid substitutions and characterized them for their effects on subcellular localization and transport activity using 10 known OCT1 substrates.

## Methods

### Populations studied

One-thousand and seventy-nine DNA samples from 53 populations worldwide were analyzed in this study: 962 samples from 52 populations worldwide were obtained from the Centre d’Etude du Polymorphisme and belong to the Human Genome Diversity Panel (HGDP-CEPH) [22]. The HGDP-CEPH panel was enriched with 117 DNA samples from healthy Caucasians from Germany [23]. The human DNA samples used in this study were completely anonymized except for geographical region of origin. The collection of the blood specimens that served as sources of DNA was performed in accordance to the Declaration of Helsinki under conditions of informed consent and with institutional review board approvals as described in details elsewhere [22, 23].

**Fig. 1:**
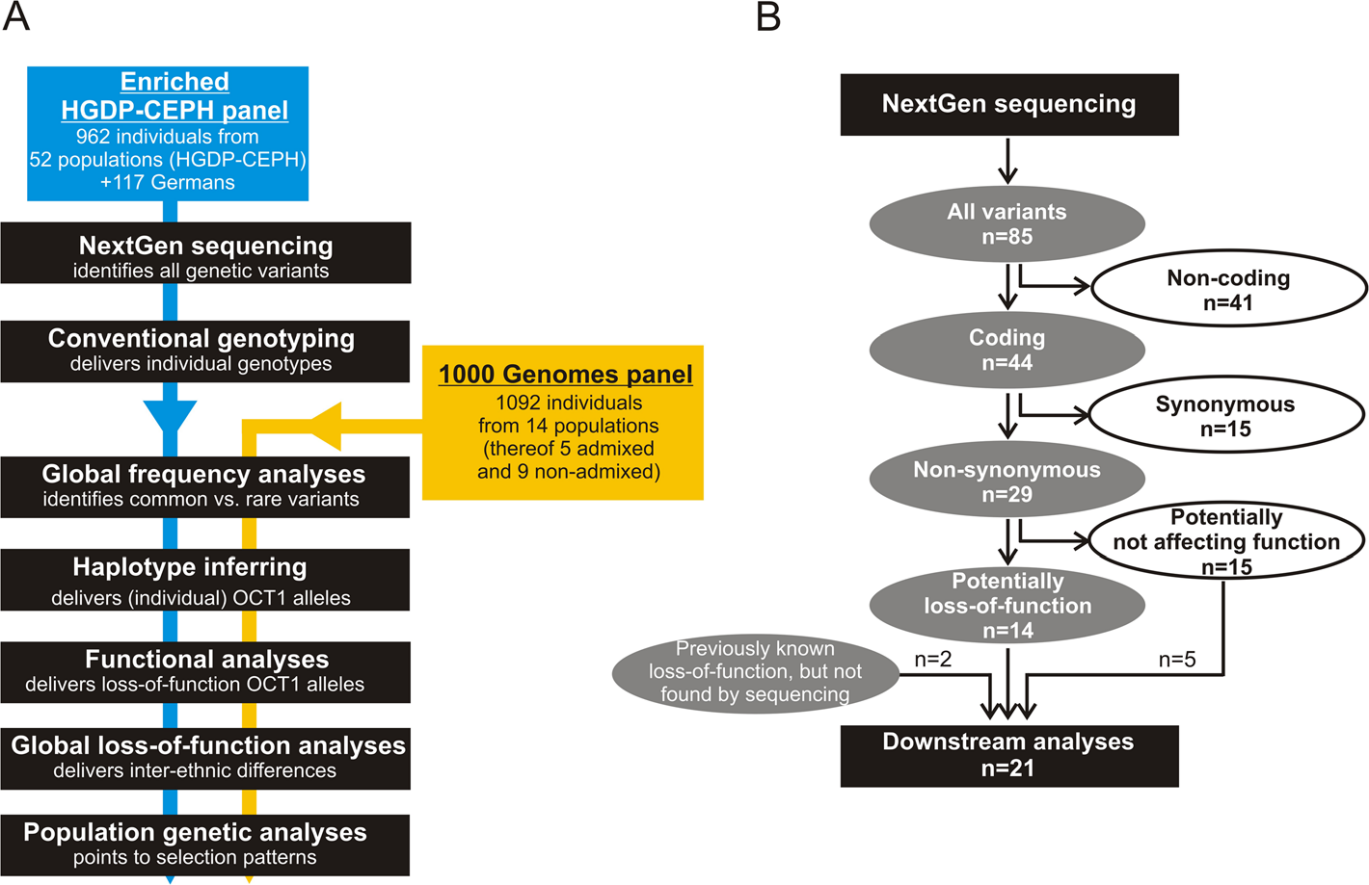
Schematic representation of the DNA samples and the genetic variants that were analyzed in this study. **a** A flowchart illustrating which analyses were performed using the enriched HGDP-CEPH panel only and which using both the enriched HGDP-CEPH panel and the 1000 Genomes panel. In the analyses that required unambiguous identification of the geographical origin of the populations (the global frequency analyses, the global loss-of-function, and the population genetic analyses) only the nine non-admixed populations of the 1000 Genomes Project were used together with the 53 populations of the enriched HGDP-CEPH panel. The highly admixed 1000 Genomes populations CLM-1 K, MXL-1 K, PRU-1 K, CEU-1 K, and ASW-1 K were omitted in these analyses. **b** A flowchart illustrating the process of selection of the 21 genetic variants that were analyzed in detail in this study. The 21 variants were selected from the variants identified by high-throughput sequencing and the variants previously suggested in the literature to cause loss of OCT1 function. In the context of the analysis pipeline presented in panel a, this diagram can be placed between the steps of ‘NextGen sequencing’ and ‘Conventional genotyping’. A detailed list of the non-coding, the synonymous, and the predicted non-deleterious variants is available in Additional file 5. We regarded variants as potentially deleterious if they were previously reported to cause a more than 50 % decrease in OCT1 activity for at least one substrate; or if they were predicted to be deleterious by at least five of eight software prediction packages used in this study. Details about the prediction software used and the prediction results are given in the text and in Additional files 3 and 6. The variants Gln97Lys and Gly220Val were not identified by sequencing of the enriched HGDP-CEPH panel, but were included in the detailed analyses as they were previously reported in the literature (Gln97Lys, [17]) or in the 1000 Genomes project (Gly220Val) and fulfilled the criteria for deleterious variants

In all the key analyses the data obtained from the 1,079 DNA samples was enriched with data from 1,092 samples from the 1000 Genome Project [24]. There was no overlap in the samples between the HGDP-CEPH and 1000 Genome panels. The analyses of the amino acid substitution frequencies, haplotype inferences, analyses of haplotype frequencies, the global analyses of loss of OCT1 activity, and the calculation of the population genetic parameters were performed using the combined HGDP-CAPH / 1000 Genomes data (Fig. 1a). Five out of the 14 populations within the 1000 Genomes project represent admixed populations and/or population of unknown geographic origin (the populations CLM, MXL, PUR, CEU, and ASW; Table 1). These populations were not used in the global analyses of loss of OCT1 activity and in the calculations of the population genetic parameters.

**Table 1:** Worldwide distribution of OCT1 alleles

World region	Population	N	chr.	Major *OCT1* alleles															
				*1	*2	*3	*4	*5	*6	*7	*8	*9	*10	*11	*12	*13	*14	*15	*16
Sub-Saharan Africa	San	6	12	91.7	0	0	0	0	0	0	8.3	0	0	0	0	0	0	0	0
	Mbuti Pygmy	13	26	76.9	7.7	0	0	0	0	0	15.4	0	0	0	0	0	0	0	0
	Biaka Pygmy	26	52	76.9	11.5	0	0	0	0	9.6	1.9	0	0	0	0	0	0	0	0
	Bantu S.W.	8	16	87.5	6.3	0	0	0	0	6.3	0	0	0	0	0	0	0	0	0
	Bantu N.E.	11	22	63.6	9.1	0	0	0	0	9.1	18.2	0	0	0	0	0	0	0	0
	Bantu (LWK-1 K)	97	194	88.1	4.1	0	0	0	0	2.6	5.1	0	0	0	0	0	0	0	0
	Mandenka	22	44	90.9	4.6	0	0	0	0	2.3	2.3	0	0	0	0	0	0	0	0
	Yoruba	22	44	95.5	0	0	0	0	0	2.3	2.3	0	0	0	0	0	0	0	0
	Yoruba (YRI-1 K)	88	176	90.3	2.3	0	0	0	0	1.7	5.7	0	0	0	0	0	0	0	0
Sub-Saharan Africa mean		293	586	84.6	5.1	0	0	0	0	3.8	6.6		0	0	0	0	0	0	0
(range)				(63.6-95.5)	(0.0-11.5)					(0.0-9.6)	(0.0-18.2)								
North Africa and Middle East	Mozabite	29	58	82.8	12.1	0	1.72	0	1.72	1.72	0	0	0	0	0	0	0	0	0
	Bedouin	46	92	72.8	9.8	10.9	0	3.26	0	2.17	1.1	0	0	0	0	0	0	0	0
	Palestinian	45	90	78.9	13.3	3.33	0	0	2.22	2.22	0	0	0	0	0	0	0	0	0
	Druze	42	84	77.4	17.9	4.76	0	0	0	0	0	0	0	0	0	0	0	0	0
North Africa and Middle East mean		162	324	78.2	13.3	4.7	0.4	0.8	1.0	1.5	0.3	0	0	0	0	0	0	0	0
(range)				(73.9-82.8)	(9.8-17.9)	(0.0-10.9)	(0.0-1.7)	(0.0-3.3)	(0.0-2.2)	(0.0-2.2)	(0.0-1.12)								
Europe	Tuscan	8	16	87.5	12.5	0	0	0	0	0	0	0	0	0	0	0	0	0	0
	Tuscan (TSI-1 K)	98	196	71.9	18.9	5.6	2.55	0.51	0	0	0.5	0	0.5	0	0	0	0	0	0
	Sardinian	28	56	69.6	21.4	1.8	5.36	0	1.79	0	0	0	0	0	0	0	0	0	0
	North Italian	14	28	89.3	7.14	3.6	0	0	0	0	0	0	0	0	0	0	0	0	0
	Iberian (IBS-1 K)	14	28	75.0	14.3	3.6	7.14	0	0	0	0	0	0	0	0	0	0	0	0
	French Basque	24	48	72.9	18.8	4.2	4.17	0	0	0	0	0	0	0	0	0	0	0	0
	French	28	56	62.5	21.4	7.14	1.79	5.36	1.79	0	0	0	0	0	0	0	0	0	0
	British (GBR-1 K)	89	178	72.5	16.3	5.06	2.25	3.37	0.56	0	0	0	0	0	0	0	0	0	0
	Orcadian	15	30	80.0	13.3	6.67	0	0	0	0	0	0	0	0	0	0	0	0	0
	German	117	234	71.4	15.8	8.12	2.14	2.56	0	0	0	0	0	0	0	0	0	0	0
	Finn (FIN-1 K)	93	186	78.5	13.4	5.38	1.61	1.08	0	0	0	0	0	0	0	0	0	0	0
	Adygei	17	34	70.6	17.6	5.88	0	2.94	0	0	0	0	2.94	0	0	0	0	0	0
	Russian	25	50	66.0	10.0	10.0	6.00	8.00	0	0	0	0	0	0	0	0	0	0	0
Europe mean		570	1,140	74.4	15.5	5.1	2.5	1.8	0.3	0	0.04		0.3	0	0	0	0	0	0
(range)				(62.5-89.3)	(7.1-21.4)	(0.0-10.0)	(0.0-7.1)	(0.0-8.0)	(0.0-1.8)		(0.0-0.5)		(0.0-2.94)						
Central Asia	Balochi	24	48	66.7	27.1	6.25	0	0	0	0	4.2	0	0	0	0	0	0	0	0
	Brahui	25	50	62.0	26.0	6.00	0	6	0	0	0	0	0	0	0	2	0	0	0
	Makrani	25	50	84.0	16.0	0	0	0	0	0	0	0	0	0	0	0	0	0	0
	Sindhi	24	48	83.3	12.5	0	0	0	0	4.17	0	0	0	0	0	0	0	0	0
	Pathan	25	50	72	26	2	0	0	0	0	0	0	0	0	0	0	0	0	0
	Kalash	23	46	73.9	19.6	6.52	0	0	0	0	0	0	0	0	0	2.17	0	0	0
	Burusho	25	50	84	6	6	0	4.00	0	0	0	0	0	0	0	0	0	0	0
	Hazara	23	46	89.1	4.35	2.17	0	0	2.17	0	0	0	0	0	0	0	2.17	0	0
	Uygur	10	20	80	15	5	0	0	0	0	0	0	0	0	0	0	0	0	0
	Yakut	25	50	92.0	0	4.00	0	0	0	0	0	4.00	0	0	0	0	0	0	0
Central Asia mean		229	458	78.7	14.8	3.8	0	1.0	0.2	0.4	0.42	0.4	0	0	0	0.4	0.2	0	0
(range)				(62.0-92.0)	(0.0-27.1)	(0.0-6.5)		(0.0-6.0)	(0.0-2.2)	(0.0-4.2)	(0.0-4.2)	(0.0-4.0)				(0.0-2.2)	(0.0-2.2)		
East Asia and Oceania	Dai	10	20	100	0	0	0	0	0	0	0	0	0	0	0	0	0	0	0
	She	10	20	90.0	0	0	0	0	0	0	0	0	0	0	10.0	0	0	0	0
	Tujia	10	20	100	0	0	0	0	0	0	0	0	0	0	0	0	0	0	0
	Yizu	10	20	100	0	0	0	0	0	0	0	0	0	0	0	0	0	0	0
	Naxi	9	18	94.4	0	0	0	0	0	0	0	0	0	5.56	0	0	0	0	0
	Tu	10	20	90.0	0	0	0	0	0	0	0	0	0	0	0	0	0	5.0	0
	Xibo	9	18	100	0	0	0	0	0	0	0	0	0	0	0	0	0	0	0
	Mongola	10	20	85.0	10.0	0	0	0	0	0	0	5.00	0	0	0	0	0	0	0
	Hezhen	9	18	100	0	0	0	0	0	0	0	0	0	0	0	0	0	0	0
	Daur	10	20	100	0	0	0	0	0	0	0	0	0	0	0	0	0	0	0
	Oroqen	9	18	100	0	0	0	0	0	0	0	0	0	0	0	0	0	0	0
	Han	45	90	100	0	0	0	0	0	0	0	0	0	0	0	0	0	0	0
	Han Beijing (CHB-1 K)	97	194	98.5	0.52	0	0	0	0	0	0	0.52	0	0.52	0	0	0	0	0
	Han South(CHS-1 K)	100	200	100	0	0	0	0	0	0	0	0	0	0	0	0	0	0	0
	Lahu	8	16	100	0	0	0	0	0	0	0	0	0	0	0	0	0	0	0
	Miaozu	10	20	100	0	0	0	0	0	0	0	0	0	0	0	0	0	0	0
	Cambodian	10	20	95.0	5.0	0	0	0	0	0	0	0	0	0	0	0	0	0	0
	Japanese	30	60	100	0	0	0	0	0	0	0	0	0	0	0	0	0	0	0
	Japanese (JPT-1 K)	89	178	100	0	0	0	0	0	0	0	0	0	0	0	0	0	0	0
	Melanesian	14	28	100	0	0	0	0	0	0	0	0	0	0	0	0	0	0	0
	Papuan	17	34	100	0	0	0	0	0	0	0	0	0	0	0	0	0	0	0
East Asia and Oceania mean		526	1,052	97.8	0.7	0	0	0	0	0	0	0.3	0	0.3	0.5	0	0	0.2	0.2
(range)				(85.0-100.0)	(0.0-10.0)							(0.0-5.0)		(0.0-5.6)	(0.0-10.0)			(0.0-5.0)	(0.0-5.0)
America	Colombian	7	14	57.1	42.9	0	0	0	0	0	0	0	0	0	0	0	0	0	0
	Karitiana	14	28	39.3	60.7	0	0	0	0	0	0	0	0	0	0	0	0	0	0
	Maya	21	42	66.7	33.3	0	0	0	0	0	0	0	0	0	0	0	0	0	0
	Pima	14	28	82.1	17.9	0	0	0	0	0	0	0	0	0	0	0	0	0	0
	Surui	8	16	6.25	93.8	0	0	0	0	0	0	0	0	0	0	0	0	0	0
America mean		64	128	50.3	49.7	0	0	0	0	0	0	0	0	0	0	0	0	0	0
(range)				(6.3-82.1)	(17.9-93.7)														
Admixed populations	Colombian (CLM-1 K)	60	120	65.8	29.2	3.33	0.83	0.83	0	0	0	0	0	0	0	0	0	0	0
	Mexican (MXL-1 K)	66	132	59.1	32.6	4.55	0	3.03	0	0.76	0	0	0	0	0	0	0	0	0
	Puerto Rican (PUR-1 K)	55	110	76.4	20.0	0.91	0	1.82	0	0	0	0	0.91	0	0	0	0	0	0
	Caucasians (CEU-1 K)	85	170	72.4	15.3	8.82	1.76	1.18	0.59	0	0	0	0	0	0	0	0	0	0
	Africans (ASW-1 K)	61	122	87.7	8.20	1.64	0.82	0	0	0.82	0	0	0	0	0	0	0	0	0.82
**Worldwide mean**		**2,171**	**4,342**	**81.0**	**12.2**	**2.14**	**0.57**	**0.65**	**0.16**	**0.68**	**0.99**	**0.14**	**0.07**	**0.09**	**0.15**	**0.07**	**0.03**	**0.07**	**0.01**
**(range)**				**(6.3-100.0)**	**(0.0-93.8)**	**(0.0-10.9)**	**(0.0-7.1)**	**(0.0-8.0)**	**(0.0-2.2)**	**(0.0-9.6)**	**(0.0-18.2)**	**(0.0-5.0)**	**(0.0-2.9)**	**(0.0-5.6)**	**(0.0-10.0)**	**(0.0- 2.2)**	**(0.2-2.2)**	**(0.0-5.0)**	**(0.0-0.6)**

*N* number of individuals analyzed, *chr.* number of chromosomes analyzed

### Massively parallel re-sequencing

Eleven regions covering the eleven *OCT1* exons and their flanking regions were resequenced using semiconductor-based massively parallel sequencing (Ion Torrent^TM^, Life Technologies, Darmstadt, Germany). The newly identified, potentially loss-of-function amino acid substitutions were validated by capillary sequencing. Conventional genotyping techniques were used to call individual genotypes of 21 amino acid substitutions. The genotypes of 16 genetic variants (Ser14Phe, Ser29Leu, Arg61Cys, Cys88Arg, Gln97Lys, Pro117Leu, Ser189Leu, Arg206Cys, Gly220Val, Thr245Met, Glu284Lys, Gly401Ser, Gly414Ala, Met420del, Ile449Thr, and Gly465Arg) were validated by single-base primer-extension reactions (SNaPshot®, Life Technologies). The genotypes of another five variants (Phe160Leu, Pro341Leu, Arg342His, Met408Val, and Arg488Met) were validated by TaqMan®-based SNP genotyping assays (Life Technologies). Sex matching was performed to exclude sample swaps and 5 % of the samples were genotyped in duplicate, showing complete concordance in the genotype calls. Arg488Met was genotyped both by TaqMan® and single-base primer-extension. The two methods showed 100 % concordance in the genotype calls. The sequencing and genotyping methods are described in details in the supplementary.

### Construction and characterization of OCT1 overexpressing cell lines

The OCT1-overexpressing HEK293 cells were generated by targeted chromosomal integration using the Flp-In System^TM^ (Life Technologies, Darmstadt, Germany). The construction and characterization of the cells overexpressing OCT1 alleles *1 (wild type), *3, *4, *5, and *6 are described in reference [25] and of the rest of the alleles in the supplementary (Additional files 1 and 2).

### OCT1 activity assays

The OCT1-mediated uptake was measured essentially as described before [3, 4, 25]. The OCT1-mediated uptake of tropisetron, debrisoquine, O-desmethyltramadol, and monocrotaline was measured using HPLC, of ASP^+^ using fluorescence spectroscopy and of radiolabelled MPP^+^, TEA^+^, morphine, metformin, and tyramine using scintillation counting. The procedures are described in details in the supplementary (Additional file 1).

### Immunofluorescence confocal microscopy and western blot analysis

The cells were trypsinized with 3.5 mL TryplLE^TM^ Express (Life Technologies) for 4 min at 37 °C and passed through a 40 μm strainer (BD Falcon, Heidelberg, Germany). Two hundred thousand cells were seeded per well of four-well chamber slides (Lab-Tek™ II, Nunc, Wiesbaden, Germany), which were pre-coated with poly D-lysine. The next day the cells were washed with phosphate buffered saline (PBS), fixed for 20 min with 4 % formaldehyde, washed twice with PBS, and permeabilized for 15 min in blocking buffer (0.5 % fetal calf serum (FCS), 0.1 % bovine serum albumin (BSA), and 0.05 % Triton X-100 in PBS). The cells were incubated with the primary antibodies diluted in blocking buffer. For primary antibodies we used mouse monoclonal anti-OCT1 (2C5; Novus Biologicals, Cambridge, UK) diluted 1:400, rabbit monoclonal anti-Na^+^/K^+^ ATPase (EP1845Y) diluted 1:200, or rabbit polyclonal anti-Calnexin (ab22595; both obtained from Abcam, Cambridge, UK) diluted 1:1000. After 1 h incubation at room temperature, the cells were washed three times for 5 min with blocking buffer and incubated with the secondary antibodies for 1 h in blocking buffer supplemented with 2.5 pg/mL DAPI (Life Technologies). The incubation was performed at room temperature with samples protected from direct light. As secondary antibodies we used Alexa Fluor® 488 goat anti-mouse or Alexa Fluor® 546 goat anti-rabbit IgG diluted 1:500 (A-11001 and A11010, respectively; Life Technologies). The cells were washed three times for 5 min with blocking buffer and once with PBS and mounted with Fluoromount-G™ (Southern Biotech; Birmingham, AL, USA). The samples were analyzed on a laser scanning microscope (LSM 710; Carl-Zeiss, Oberkochen, Germany) and the images were processed using ImageJ software v. 1.47 (NIH, Bethesda, MD, USA) with additional contrast adjustment for each channel.

The western blot was performed with total protein extracts of HEK293 cells stably transfected with the different OCT1 variants. The detection was carried out using mouse monoclonal anti-OCT1 (2C5; Novus Biologicals, Cambridge, UK) and monoclonal anti-GAPDH (6C5; Zytomed, Berlin, Germany) as primary antibodies and horseradish peroxidase-conjugated anti-mouse antibody (BioMol, Hamburg, Germany) as secondary antibodies. Procedure is described in detail in the supplementary.

### Computational analyses

Individual *OCT1* haplotypes were inferred using PHASE version 2.1 [26]. Ten independent runs with different seeds (2, 1,536, 2,936, 3,123, 4,957, 5,283, 6,757, 7,992, 8,633, 9,045) were performed to exclude seed-biased assignments.

Effects of the non-synonymous variants in *OCT1* on protein function were predicted using eight different tools: PROVEAN version1.1.3, SIFT version 1.0.3, PolyPhen-2 version 2.2.2, MutPred version 1.2, Mutation t@sting version 2, SNPs3D, SNAP, and PhD SNP (for details see Additional file 3).

Analysis of molecular variance (AMOVA) was performed on three levels, within population, among populations within each geographic region and among the geographic regions. The calculations were done using Arlequin v3.5.1.2 [27] for the 62 populations in six world regions worldwide. The admix populations from the 1000 Genome Project were excluded from the analysis. Data were entered as DNA sequence data (haplotypic) as the input method for the 16 haplotypes, while we used the allele frequency input method for the phenotypes. Phenotypes were coded as 0, 1, and 2 referring to zero, one, and two active OCT1 alleles, respectively.

The calculations of the average nucleotide diversity (π), the estimation of the population mutation parameter (θ), and the test for neutrality (Tajima’s D) were performed using Arlequin v3.5.1.2 [27]. The values for π and θ were normalized to the size of the coding region of the *OCT1* gene. All parameters were calculated independently for the six major world regions, and the nine admixed population of the 1000 Genomes Project were excluded from the analysis.

## Results

### Global genetic variability in OCT1

Applying semiconductor-based massively parallel sequencing, we analyzed the global genetic variability in the coding regions of *OCT1*. We resequenced 2,770 bp of the *OCT1* gene, including the complete coding region, the untranslated regions and the intron-exon junctions (Additional file 4). We obtained more than 7.1 million mapped sequencing reads. The average coverage depth was 173-fold per sample (range 17 to 402) with 977 samples (91.3 %) having average coverage above 50-fold. The complete sequencing dataset and individual genotypes were deposited and are freely available in the HGDP-CEPH Database [28].

**Fig. 2:**
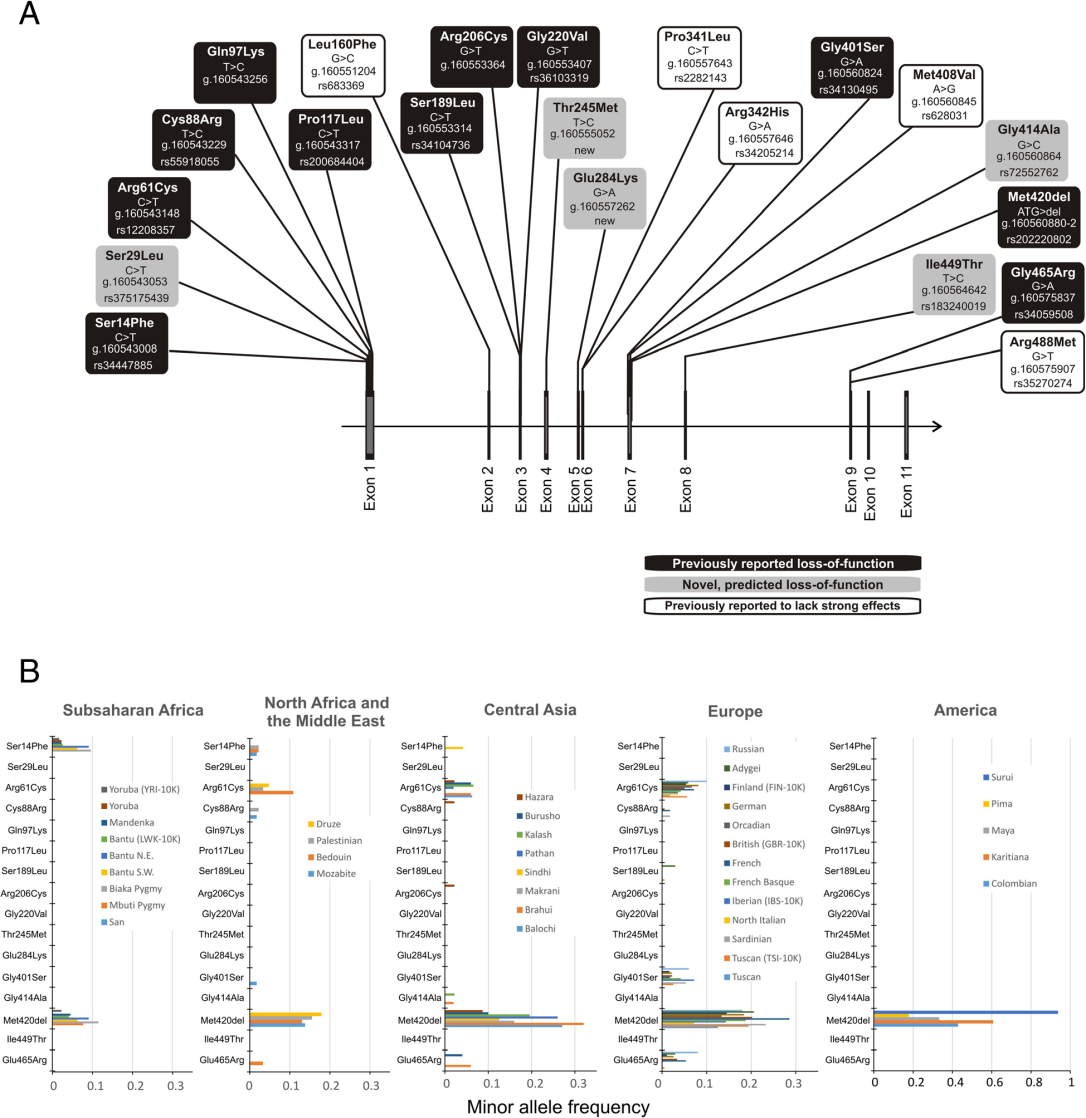
Non-synonymous *OCT1* polymorphisms causing potentially functional amino acid substitutions, and their distribution in different world regions. **a** The localization of the 21 polymorphisms analyzed in details in this study. Substitutions previously reported in the literature to have strong functional effects on OCT1 activity are shown in black (reported loss of function), substitutions predicted to affect OCT1 activity are shown in gray (predicted loss-of-function), and substitutions previously reported to not affect or to cause less than 50 % reduction of OCT1 activity are shown in white (reported to lack strong effects). The polymorphisms are designated with the amino acid substitutions that they cause and the codon that is affected. Their rs-number in the dbSNP database (if available) and their location on chromosome 6 according to the human genome assembly hg19 are also given. **b** Minor allele frequencies of the 16 known or predicted loss-of-function OCT1 polymorphisms. Shown are 39 populations from Sub-Saharan Africa, North Africa and the Middle East, Central Asia, Europe, and America. The 39 populations shown include 35 populations from the CEPH human genome diversity panel and five from the 1000 Genome Project [24] (designated with 1 K). The populations CEU, ASW, MXL, PUR, and CLM from the 1000 Genomes Project were omitted from these analyses due to their highly admixed structure and/or inability to be allocated to a defined world region

We identified a total of 85 variants (a detailed list of all variants is available in Additional file 5). Thereof 44 variants were within the coding region of OCT1 and 29 of them caused amino acid substitutions. We selected 21 amino acid substitution variants for our subsequent analyses (see Fig. 1b for the selection strategy). Included were 14 amino acid substitutions that may affect OCT1 activity (Fig. 2a): nine variants previously known to cause loss of OCT1 activity (Ser14Phe, Arg61Cys, Cys88Arg, Pro117Leu, Ser189Leu, Arg206Cys, Gly401Ser, Met420-deletion, Gly465Arg) [2–5, 7, 10, 11, 17] and five variants predicted to cause loss of OCT1 function (Ser29Leu, Thr245Met, Glu284Lys, Gly414Ala, Ile449Thr). To predict loss of OCT1 function we used eight independent prediction tools. Variants that were predicted to be deleterious by at least five of the eight prediction tools were regarded as potential loss-of-function variants (Additional file 6). We also genotyped two variants, Gln97Lys and Gly220Val, which were not identified in our population by massively parallel sequencing. However, Gln97Lys was previously reported in the literature to strongly affect OCT1 activity [17], while Gly220Val was observed in the 1000 Genomes project and was predicted to be deleterious (Additional file 6). Furthermore, we analyzed five amino acid substitutions (Leu160Phe, Pro341Leu, Arg342His, Met408Val, and Arg488Met) that were known to either not affect or to cause less than 50 % reduction in OCT1 activity [2, 10, 11, 20, 21]. We included these variants for two reasons. First, we wanted to obtain information about the effects of these variants on the uptake of a much broader spectrum of substrates than previously tested. Second, we aimed to identify common amino acid substitutions that do not affect OCT1 activity as a control in our population genetic analyses.

The methionine_420_ deletion (Met420del) was the most commonly observed loss-of-function variant among the 1,079 individuals from 53 populations analyzed (worldwide minor allele frequency of 14.1 %). The other common loss of function variants were Arg61Cys (3.2 %), Gly465Arg (1 %), Ser14Phe (0.8 %), and Gly401Ser (0.7 %). The variants Ser29Leu, Cys88Arg, and Pro117Leu were observed only in two, eight, and four unrelated individuals, respectively. The variants Ser189Leu, Arg206Cys, Thr245Met, Glu284Lys, and Ile449Thr were observed only in single individuals. The variant Gly220Val was observed in a single individual in the 1000 Genomes sample and was missing in the HGDP-CEPH samples. The variant Gln97Lys was not observed in either sample set.

Among the loss-of-function OCT1 variants, Met420del was the only ubiquitous OCT1 variant that was observed on all continents (Fig. 2b). The Ser14Phe, Arg342His, and Arg488Met variants were frequent in Africa and occasionally observed in the Middle East and Central Asia, but were completely missing in Europe and America. In contrast, the variants Arg61Cys and Gly465Arg were frequent in Europe, occasionally observed in the Middle East and Central Asia, but completely missing in Africa and America. The rare variants Ser29Leu, Pro117Leu, Arg206Cys, Thr245Met, Glu284Lys, and Ile449Thr were observed only in Asians populations. Among the variants that were not expected to strongly affect OCT1 function, Met408Val was the most frequent. Pro341Leu was the second-most frequent variant. It was observed in all the world regions, but was more common in East Asia.

Comparison with the primate OCT1 sequences showed that, with the exception of serine_14_ and arginine_488_, all the major alleles in humans were also the ancestral alleles (Additional file 7). The primates have phenylalanine at codon 14 and methionine at codon 488 while the major allele in humans is serine_14_ and arginine_488_, respectively.

### OCT1 alleles and their functional characteristics

**Fig. 3:**
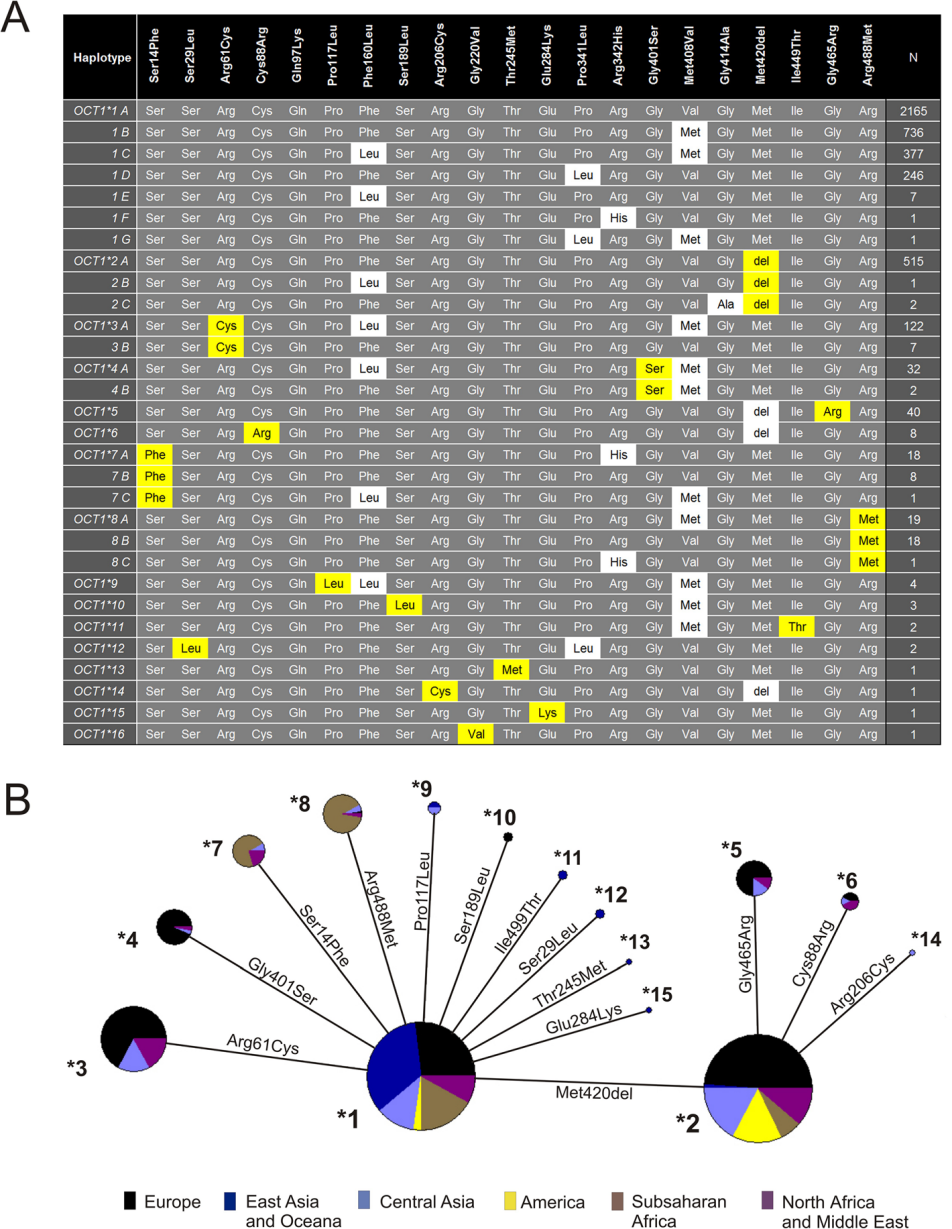
*OCT1* alleles and their global distribution. **a** Shown are the haplotype combinations constituting the 16 major and 14 sub-alleles of OCT1 observed in the worldwide genetic analyses. As major we defined alleles carrying amino acid substitutions (highlighted in yellow) that cause a more than 50 % decrease or 50 % increase of OCT1 activity in comparison to the reference OCT1*1 allele for at least one substrate tested. The sub-allele of a major allele differs by amino acid substitutions (highlighted in white) that do not affect OCT1 activity. For details about variants affecting or not affecting OCT1 activity see Fig. 4 and the results section in the text. **b** Cladogram illustrating the relations between the major *OCT1* alleles and their worldwide distribution. Different world regions are indicated in colors. Pie size represents the global allele frequency. The amino acid substitutions that distinguish between the alleles are shown. Allele OCT1*16 is missing from the cladogram as it was observed only in admixed population from the 1000 Genomes Project and could not be assigned to any world region. The cladogram was generated using the median joining algorithm of the NETWORK software version 4.6 (Fluxus Technology Ltd.)

**Fig. 4:**
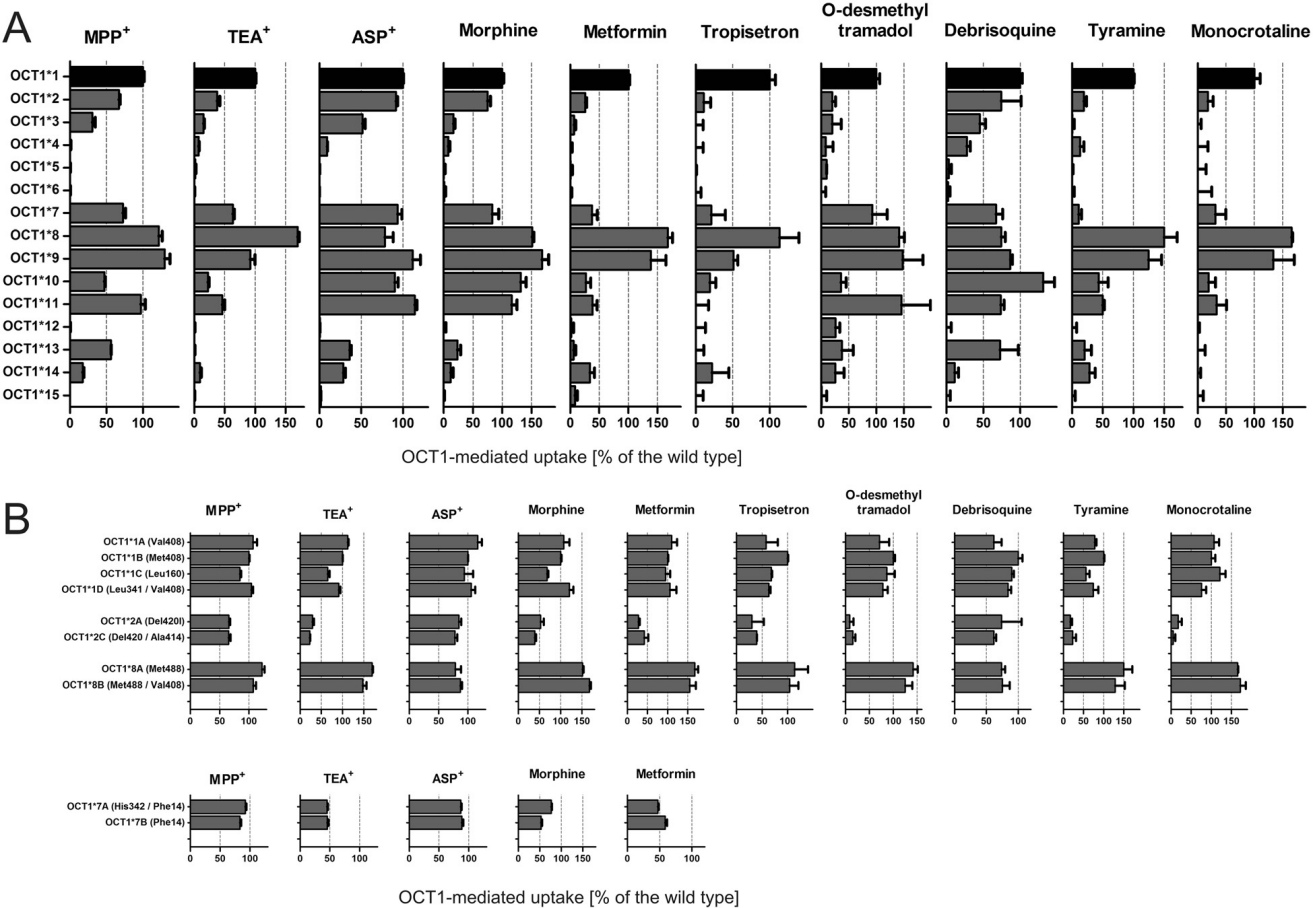
Comparative analyses of the activity of the different OCT1 alleles using 10 different OCT1 substrates. Shown are the effects of the major *OCT1* alleles (**a**) and the six most common sub-alleles (**b**). The allele activity was determined with cellular uptake measurements in HEK293 cells overexpressing different *OCT1* alleles. The uptake was measured after incubation for 2 min with 10 μM MPP^+^, 5 μM TEA^+^, 5 μM ASP^+^, 1 μM morphine, 5 μM metformin, 10 μM tyramine, or 1 μM monocrotaline, incubation for 1 min with 1 μM debrisoquine or 1 μM O-desmethyltramadol, or for 3 min with 1 μM tropisetron. OCT1-mediated uptake was calculated by subtracting the uptake in HEK293 cells transfected with the empty pcDNA5/FRT vector from the uptake in the cells transfected with the different *OCT1* alleles. The results were represented as a percent of the uptake in the cells transfected with the OCT1*1 allele (shown in black). The values for the uptake of morphine and tropisetron by alleles *3, *4, *5, and *6 are from our previously published studies [3] and [5], respectively. The comparison between OCT1*7A and OCT1*7B alleles (b) were made after transient transfection. All the remaining experiments were performed after stable transfection in HEK293 cells. Shown are mean and standard error of the means of three or more independent experiments

Using haplotype inferring of the extended HGDP-CEPH and the 1000 Genome Project data we identified 30 haplotypes (Fig. 3a). The haplotypes were grouped to 16 major alleles (designated *1 to *16) and 14 sub-alleles (designated with a capital letter after the major allele number, for example, *1A, *1B, and so on). As sub-alleles we defined alleles that do not showed more than 50 % reduction or 50 % increase of the activity compared to the corresponding major allele with any of the substrates tested (for details see Fig. 4 and the text below). The non-synonymous variants causing substantial changes in OCT1 activity the variants Cys88Arg, Arg206Cys, and Gly465Arg were not observed alone, but in combination with the Met420del variant (Fig. 3b).

Whereas the allele structure was the same in all populations studied here, the frequencies of the alleles varied substantially among the world regions. The global population frequencies of the major *OCT1* alleles are shown in Table 1 and detailed information regarding the allele frequencies of the sub-alleles is given in Additional file 8. Next to the major OCT1*1 allele, OCT1*2 was the only evolutionary old major allele observed in all populations. OCT1*2 was the only loss-of-function allele that was observed in Native Americans. Alleles *3 to *8 were region-specific: *3 was observed in Europe, the Middle East and Central Asia, *7 and *8 were observed in Africa, the Middle East, and occasionally in Central Asia. Alleles *9 to *16 were very rare on a global scale (allele frequencies of below 0.5 %). However, in some specific populations their frequencies may be much higher, for example, the OCT1*12 allele frequency reached 10 % in the population of She Chinese (Table 1).

Next, we performed functional analyses of 19 of the 21 non-synonymous variants. Two non-synonymous variants, Gln97Lys and Gly220Val, were omitted from the analyses. Gln97Lys was not observed in any of the samples studied here. Gly220Val was observed only in a single individual from an admixed population that could not be used in the global loss of OCT1 function analyses. The 19 variants tested constituted 22 alleles: the 15 major alleles and the seven most common sub-alleles. The 22 alleles tested represent a total of 4,321 of the 4,342 chromosome 6 copies of the individuals analyzed in this study (Fig. 3a). We characterized the functional activity of the *OCT1* alleles in stably transfected HEK293 cells. The transfection resulted in a strong and constant overexpression of the mRNA of the different *OCT1* alleles (Additional file 2). We measured the ability of the isoforms encoded by the different alleles to transport the model OCT1 substrates 1-methyl-4-phenylpyridinium (MPP^+^), tetraethylammonium (TEA^+^), and 4-(4-(dimethylamino)styrl)-N-methyl-pyridinium (ASP^+^), the drugs debrisoquine, morphine, metformin, tropisetron and O-desmethyltramadol (the active metabolite of tramadol), and the naturally occurring substances tyramine and monocrotaline (Fig. 4a). Tyramine is a biogenic amine suggested to be an OCT1 substrate [29] and was transported by OCT1*1 in our hands with a K_M_ of 94.7 μM and V_MAX_ of 381 pmol/mg protein/min (Additional file 9).

The overexpression of wild-type OCT1 (OCT1*1) resulted in an increase in the uptake of the substances tested by at least 1.9-fold (range 1.9- to 24.1-fold, Additional file 10). The non-synonymous variants Phe160Leu, Pro341Leu, Arg342His, Met408Val, and Gly414Ala did not cause a more than 50 % decrease or 50 % increase in OCT1 activity with any of the substrates tested (Fig. 4b). Therefore haplotypes carrying these variants were designated as sub-alleles (Fig. 3).

In addition to the sub-alleles, which did not show strong changes to the OCT1*1 activity, we identified two gain-of-function *OCT1* alleles (the major alleles OCT1*8 and OCT1*9) and 11 loss-of-function alleles (the major alleles OCT1*2 to *7 and OCT1*10 to *15; Fig. 4a). The allele OCT1*8 (characterized by the presence of the Arg488Met substitution) showed a more than 50 % increase in the uptake of TEA^+^, morphine, metformin, and monocrotaline. The allele OCT1*9 (Pro117Leu) showed 68 % increase in the uptake of morphine.

The major alleles showing loss of activity could be also separated in three independent groups. The alleles *5 (a combination of Gly465Arg and Met420del), *6 (a combination of Cys88Arg and Met420del), *12 (Ser29Leu), and *15 (Glu284Lys) showed complete loss of OCT1 activity independent of the substrate used (Fig. 4). The alleles *3 (Arg61Cys), *4 (Gly401Ser), and *14 (a combination of Arg206Cys and Met420del) showed strong, but not complete, loss of activity also independent of the substrate. In contrast, the alleles *2 (Met420del), *7 (Ser14Phe), *10 (Ser189Leu), *11 (Ile449Thr), and *13 (Thr245Met) showed substrate-specific loss of activity. The transport activity of alleles *2, *7, *10, and *11 was reduced to 0 % of the OCT1*1 activity with some of the substrates tested, but was more than 90 % for others (Fig. 4a).

We analyzed the combined effects of the Met420del and Met408Val variants on OCT1 activity in terms of affecting subcellular localization and activity of OCT1. Neither the presence of the Met420 deletion, nor Met408Val affected the correct localization of OCT1 at the plasma membrane (Additional file 11). The presence of Met420del resulted in a substrate-specific loss of OCT1 activity. The Met408Val substitution did not cause differences in the transport of metformin, tyramine, or debrisoquine. However, a small but significant increase of morphine uptake was observed when methionine_408_ was substituted to valine suggesting that when morphine is used as a substrate OCT1*2 allele should be regarded as a reduced activity allele (and not as a complete loss of activity allele as suggested earlier [3]).

We analyzed the ability of model substrates to predict the ability of the alleles that conferred substrate-specific loss of OCT1 activity (*2, *7, *10, *11, and *13) to transport the clinically relevant drugs metformin and morphine (Additional file 12). There was no single model substrate that was able to accurately predict the effect of OCT1 variants on the uptake of both drugs. Whereas morphine uptake correlated better with ASP^+^ uptake, metformin uptake correlated better with TEA^+^ uptake. These results underline the substrate-specific effects of variants *2, *7, *10, *11, and *13 with the consequence that there will not be a single model substrate completely reflecting the effects of these genetic variants on OCT1 activity*.*

**Fig. 5:**
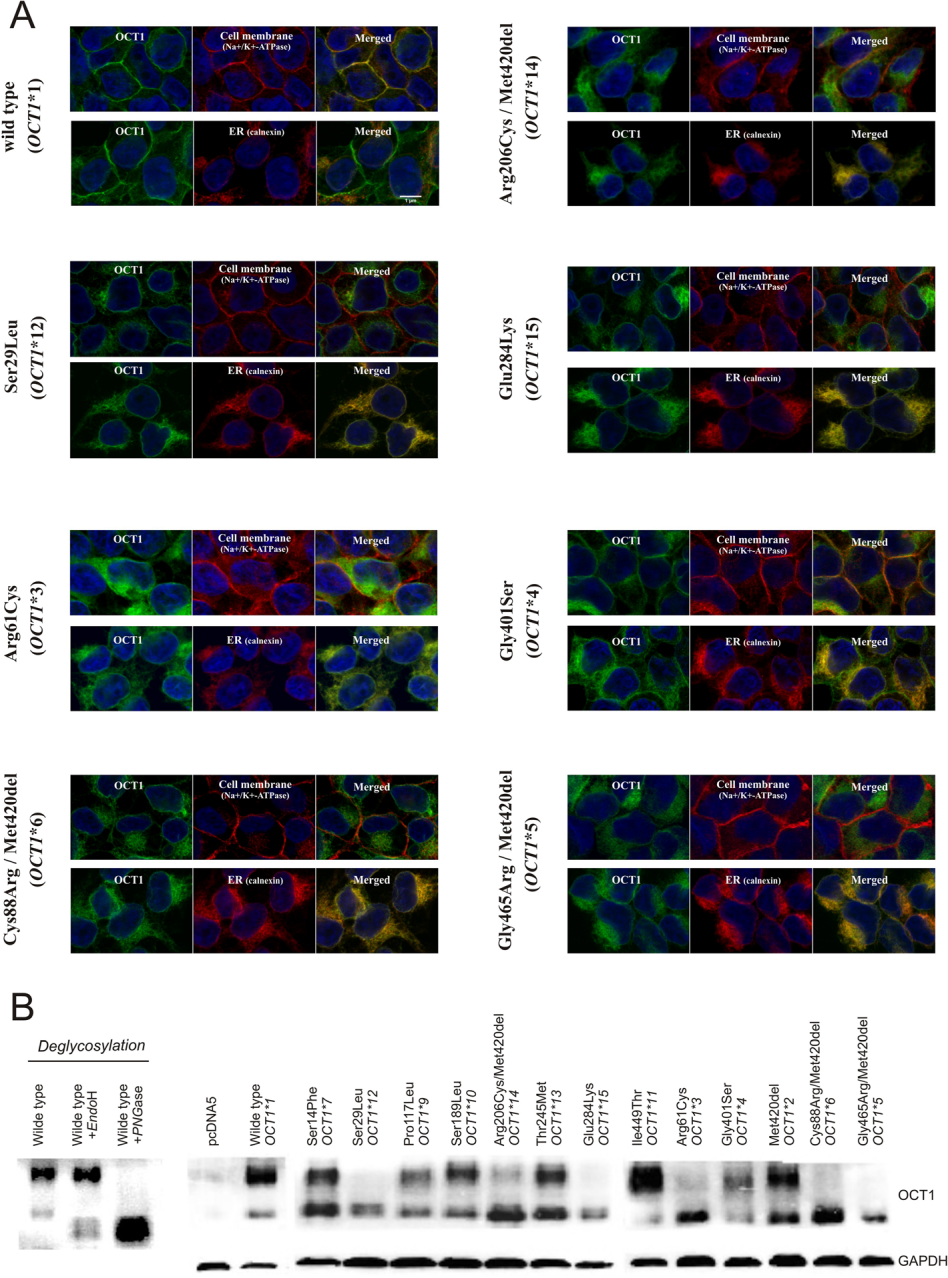
Subcellular localization and glycosylation patterns of the isoforms encoded by the major *OCT1* alleles. **a** The subcellular localization of the OCT1 isoforms showing that the major cause for substrate-wide loss of OCT1 activity is improper subcellular localization. Shown are the OCT1-overexpressing HEK293 cells stably transfected with alleles causing substrate-wide loss of OCT1 function, and the HEK293 cells expressing the wild type (OCT1*1) as a reference. The subcellular localization was analyzed with confocal microscopy after immunocytochemical staining for OCT1 (green). The exact OCT1 localization was analyzed using co-staining with Na^+^/K^+^ ATPase (red, upper part) as a marker for the plasma membrane and calnexin (red, lower part) as a marker for the endoplasmic reticulum. The immunocytochemical analyses of the sub-alleles and the alleles causing gain or substrate-specific loss of OCT1 function are shown in Additional file 13. **b** Western blot analyses of total cellular protein illustrating differences between the glycosylation patterns of OCT1 isoforms correctly localized in the plasma membrane and those retained in the endoplasmic reticulum. OCT1 was detected as a double signal of a close to 70 kDa large *PNGase*F-sensitive and *Endo*H-resistant glycosylation form and a 50 kDa *Endo*H-sensitive glycosylation form. Glyceraldehyde-3-phosphate dehydrogenase (GAPDH) was used as a loading control

Next we analyzed the subcellular localization of the OCT1 isoforms encoded by the 14 major alleles and the four most common sub-alleles. Most isoforms with substrate-wide loss of function failed to localize in the plasma membrane (Fig. 5a). Co-localization with calnexin suggested retention in the endoplasmic reticulum. Therefore, lack of correct localization was the major reason for substrate-wide loss of OCT1 activity. The only exception was the allele OCT1*4 (Gly401Ser). OCT1*4 was correctly localized in the plasma membrane, but had between 0 and 28 % of the wild type activity in the substrates tested (Fig. 4a). In contrast, the isoforms with substrate-dependent loss of activity showed normal cellular localization (Additional file 13).

The lack of plasma membrane localization correlated with an altered glycosylation pattern of OCT1 (Fig. 5b). Western blot analyses showed that the variants that were not properly localized in the plasma membrane (OCT1*5, *6, *12, *15, and partially *3 and *14) lacked their *PNGase*F-sensitive and *Endo*H-resistant glycosylation, but kept their *Endo*H-sensitive glycosylation.

### Global variations in loss of OCT1 activity

Finally and most importantly, we analyzed the frequency of genotypically predicted loss of OCT1 activity worldwide. To this end we categorized the individuals as carriers of two, one or zero completely active OCT1 alleles. We regarded the OCT1 alleles *1, *8, and *9 as completely active. The remaining major alleles *2, *3, *4, *5, *6, *7, *10, *11, *12, *13, *14, and *15 we regarded as loss of activity as they show more than 65 % reduction in the OCT1 activity with at least one substrate tested (Fig. 4a).

Between Europeans there were only minor differences in frequency of loss of OCT1 activity (Additional file 14). On average 53 % of the Europeans carried two, 38 % one and 8 % zero active OCT1 alleles. These values were similar to those reported before [4, 10, 11]. A slightly higher frequency of loss of OCT1 activity was observed in Russia and a slightly lower frequency in Northern Italy, but this may be due to the small size of the population samples (25 and 14 individuals, respectively).

**Fig. 6:**
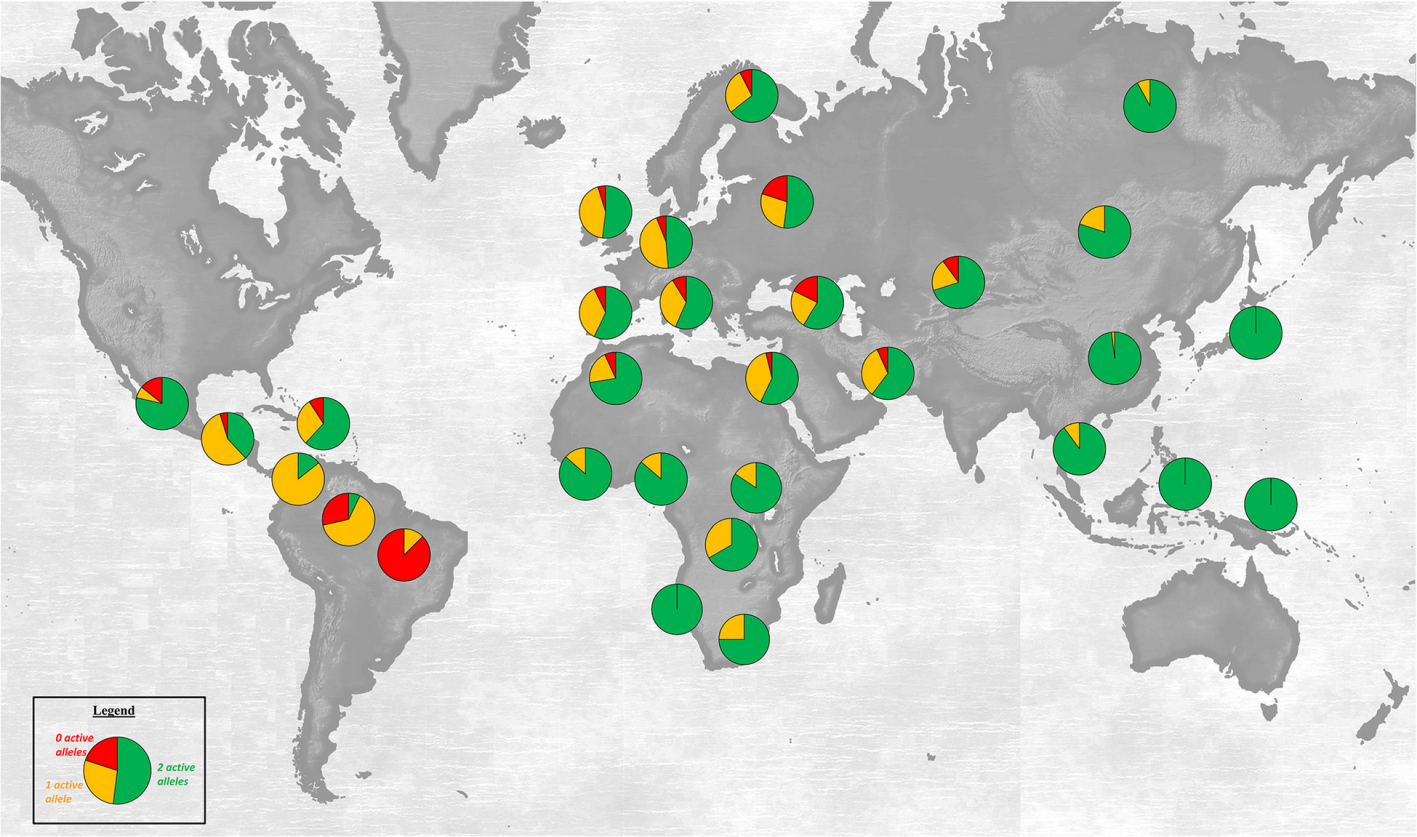
World map of genetically determined loss of OCT1 activity. Frequencies of carriers of two (green), one (orange), and zero (red) completely active *OCT1* alleles are shown for different regions around the world. The major alleles OCT1*1, *8, and *9 were regarded as completely active

Worldwide, we observed strong variations in the frequency of loss of OCT1 activity (Fig. 6). Whereas all individuals from Japan and Oceania carried two completely active *OCT1* alleles, the majority of the Surui Indians carried two completely inactive *OCT1* alleles. Out of eight Surui Indians analyzed, one (13 %) carried only one and the remaining seven (87 %) completely lacked any active *OCT1* alleles. Similarly to the individuals from Japan, more than 98 % of the Han Chinese, the major population in China, carried two completely active OCT1 alleles and the remaining less than 2 % carried one (Additional file 14). Similar to the Surui, other South American Indians had high frequencies of loss of OCT1 activity, for example, only 7 % of the Karitina Indians carried two completely active OCT1 alleles, 64 % carried one, and 29 % lacked any completely active OCT1 alleles.

Analyses of molecular variance (AMOVA) showed strong variability among the regions of the world at haplotype level and even stronger at phenotypic level (10.5 % and 18.7 %, respectively; Table 2). Within the different geographic regions the highest variability among the populations was observed by the Native Americans (36.3 % at phenotypic level) compared to only 1.3 % by the European populations.

**Table 2:** Global analysis of molecular variance in OCT1 gene

	Number of regions	Number of populations	Haplotypes				Phenotypes		
			Within populations	Among populations within regions	Among regions		Within populations	Among populations within regions	Among regions
World	1	62	89.2	10.8			79.9	20.1	
World	6	62	87.4	2.1	10.5		76.9	4.4	18.7
Sub-Saharan Africa	1	9	97.6	2.4			91.7	8.3	
North Africa and Middle East	1	4	99.9	0.1			99.5	0.5	
Europe	1	13	100.0	0.0			98.7	1.3	
Central Asia	1	10	96.2	3.8			91.5	8.5	
East Asia and Oceania	1	21	94.9	5.1			89.5	10.5	
America	1	5	75.6	24.4			63.7	36.3	

**Fig. 7:**
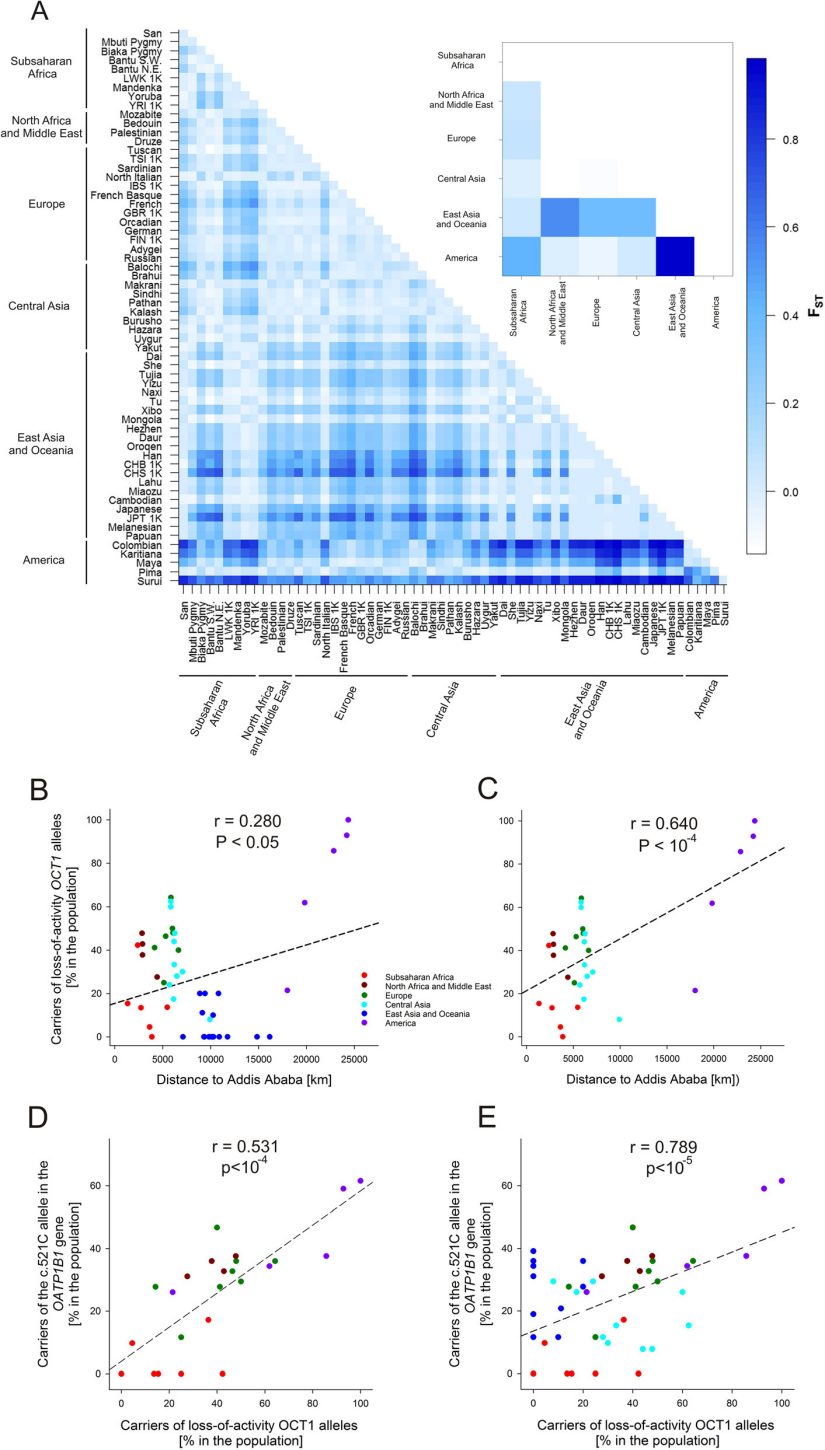
Analyses of the variations in the frequencies of loss of OCT1 activity. **a** Pairwise comparison of the divergences between the populations and between the world regions (the upper panel). The divergences between the populations were shown in terms of pairwise fixation indexes (F_ST_). The deep blue color corresponds to high divergence between the populations / regions (high F_ST_ values). We analyzed 62 populations from the 1000 Genomes Project (designated with 1 K) and HGDP-CEPH project (the rest). The populations were stratified in world regions as shown in Table 1. The populations CEU, ASW, MXL, PUR, and CLM from the 1000 Genomes Project were omitted from these analyses due to their highly admixed structure and/or inability to be allocated to a defined world region. **b** and **c** Correlation analyses between the loss of OCT1 and geographic distance to Addis Ababa. Shown are all HGDP-CEPH populations (**b**) and populations outside East Asia and Oceania (**c**). The distances to Addis Ababa are according to the previously published ones [37]. Shown are Pearson’s correlation coefficients. The number of populations analyzed were 50 in (**b**) and 32 in (**c**). **d** and **e** Correlation analyses between the loss of OCT1 and of OATP1B1 activity in HGDP-CEPH populations. Shown are all populations (**d**) and populations outside Asia and Oceania (**e**). Loss of OATP1B1 activity is represented as the presence of the C-allele of the c. 521 T > C polymorphism. The carrier of the C-allele were estimated from the population data of Pasanen *et al.* [30] assuming Hardy-Weinberg distribution of the genotypes within each population. Shown are Pearson’s correlation coefficients. The number of populations analyzed were 52 and 24 in (**d**) (**e**), respectively

Pairwise F_ST_ values analyses showed strong divergence in the loss of OCT1 activity between the America and the East Asia populations (F_ST_ of 0.76, Fig. 7a). Similar results were obtained by analyzing the *OCT1* genetic variability at the genetic level without regarding the functional effects of the haplotypes (F_ST_ of 0.72, Additional file 15). There was only limited positive correlation between the frequency of loss of OCT1 activity and the geographic distance to Addis Ababa, which may be regarded as a starting migration point of the modern humans according to the Out-of-Africa model (r = 0.28, *P* <0.05, Fig. 7b). However, the correlation was much stronger when the populations from East Asia and Oceania were excluded from the analyses (r^2^ = 0.640, *P* <10^−4^, Fig. 7c).

In addition, we took advantage of the available genotypes of the HGDP-CEPH individuals for another polymorphic drug transporter OATP1B1 [30] and the metabolizing enzyme CYP2D6 [31] and analyzed the correlation between loss of OCT1 activity and loss of OATP1B1 or CYP2D6 activity. We regarded the presence of the c.521C (Ala174) allele as a loss of OATP1B1 activity. The c.521C allele was reported both *in vitro* and *in vivo* to be the common polymorphism that most strongly affects OATP1B1 activity [32–34]. The frequency of loss of OCT1 activity correlated with the frequency of loss of OATP1B1 activity (r = 0.531, *P* <10^−4^, Fig. 7d). The correlation was much stronger when the Asian and Oceanian populations were excluded from the analyses (r^2^ = 0.789, *P* <10^−5^, Fig. 7e). We regarded the presence of any of the nonfunctional *CYP2D6* alleles, CYP2D6 *3, *4, *5, or *6, as a loss of CYP2D6 activity. No correlation was observed between the loss of OCT1 activity and loss of CYP2D6 activity (data not shown).

Next we performed classical population genetic analyses to test for neutrality of the observed loss of OCT1 function. We analyzed the region-specific variation in nucleotide diversities (π) of the amino acid substitutions in OCT1 and compared them with the expected average heterozygosity (also known as population mutation parameter θ; Table 3). The observed nucleotide diversity in the different world regions was on average 5.88 × 10^−4^, which was very close to the previously reported value (5.11 × 10^−4^, [16, 35]). The nucleotide diversity varied between 3.6 × 10^−4^ and 7.9 × 10^−4^ among the world regions (Table 3).

**Table 3:** Population genetic statistics of the OCT1 gene for the different world regions

World region	π (×10^−4^)					θ (×10^−4^)				Tajima’s D		
	All	LOF	Non-LOF	π_LOF_ / π_non-LOF_		All	LOF	Non-LOF		All	LOF	Non-LOF
Sub-Saharan Africa	4.91	0.85	4.06	0.21		5.19	1.73	3.46		−0.096	−0.601	0.271
North Africa and Middle East	5.73	2.59	3.15	0.82		9.45	5.67	3.78		−0.869	−1.040	−0.280
Europe	7.93	3.06	4.87	0.63		8.52	4.65	3.87		−0.140	−0.568	0.400
Central Asia	7.60	2.36	5.24	0.45		11.73	5.42	6.32		−0.803	−1.042	−0.330
East Asia and Oceania	5.48	0.16	5.32	0.03		7.99	4.79	3.20		−0.621	−1.638*	0.976
America	3.63	2.76	0.87	3.18		4.25	1.06	3.18		−0.268	1.675	−1.204

LOF, loss-of-function. Included are all variants causing a more than 50 % reduction in OCT1 activity for at least one substrate tested, that is, Ser14Phe, Ser29Leu, Arg61Cys, Cys88Arg, Ser189Leu, Arg206Cys, Thr245Met, Glu284Lys, Gly401Ser, Met420del, Ile449Thr, and Gly465Arg; non-LOF, non-loss-of-function. Included are all variants that did not affect or caused an increase in OCT1 activity, that is, Pro117Leu, Phe160Leu, Pro341Leu, Arg342His, Met408Val, Gly414Ala, and Arg488Met

*P <0.01

We took advantage of the detailed functional analyses that we performed in this study and analyzed separately the nucleotide diversity for the amino acid substitutions causing loss of OCT1 function (π_LOF_) and the ones not causing loss of function (π_non-LOF_). We observed strong, more than 10-fold variations in π_LOF_ among the different world regions (Table 3). By far the lowest π_LOF_ was observed in East Asia and Oceania (0.16 × 10^−4^). In addition, the ratio of π_LOF_ to π_non-LOF_ was extremely low for East Asia and Oceania (0.03 compared to an average of 0.89 for the whole world regions, Table 3). Furthermore, the observed loss-of-function nucleotide diversity in East Asia and Oceania was significantly lower than the expected value (θ of 4.8 × 10^−4^; Tajima’s D of −1.64, *P* <0.01). This indicates the presence of purifying selection for loss-of-function OCT1 variants in this region. In contrast, the ratio of π_LOF_ to π_non-LOF_ variants in Native Americans was very high, but the difference between the observed and expected loss-of-function nucleotide diversity was not significant (Table 3).

## Discussion

The comprehensive population and functional genetic analyses performed in this study revealed strong variability in the frequency of genetically-determined loss of OCT1 activity among populations around the world. Whereas in East Asia lack of OCT1 activity was very rare, lack of OCT1 activity was very common in some native South American populations, who were mostly carriers of the OCT1*2 allele. Depending on the substrate, more than 80 % of the Surui Indians completely lack OCT1 activity and the remaining less than 20 % have only one active *OCT1* allele. The genetic variability of OCT1 among different world regions was much higher compared to other genes involved in the detoxification of xenobiotics and known for their high genetic variability like CYP2D6. Whereas a previously published analysis of genetic variability of CYP2D6 in the HGDP-CEPH panel found only 6.5 % phenotypic variability among world regions [31], here we observed 18.7 % variability for OCT1 (Table 2).

The observed strong population-specific loss of OCT1 activity may reflect in population-specific differences of pharmacokinetics and therapeutic efficacy of some commonly used drugs. Thus far, most clinical studies on *OCT1* polymorphisms have been performed in Caucasians. Using the data from this study, the results may be extrapolated to other populations worldwide. Numerous drugs including metformin, morphine, tramadol, and tropisetron have been suggested to depend on OCT1 transport to be metabolized or to exert their pharmacological effects [2–5]. Furthermore, genetically determined loss of OCT1 activity was found to correlate with variations in the pharmacokinetics and the efficacy of these drugs [2–5, 12]. Based on the results of this study it should be expected that populations from East Asia and Africa will have generally lower and less inter-individually variable plasma concentrations of these drugs. Indeed, Sadhasivam *et al.* reported lower plasma concentrations of morphine in the children of African Americans, compared to white Americans [36] and suggested that this may be due to the more common loss of OCT1 activity in Caucasians compared with Africans [12]. This hypothesis is supported by our data. Our analyses showed that in Sub-Saharan Africa only 5 % of the population carry alleles causing a reduction in OCT1-mediated uptake of morphine, in contrast to 25 % in Europe (Table 1 and Fig. 4a).

The highly variable frequency of loss-of-function OCT1 polymorphisms suggests the existence of selection pressure for losing (or maintaining) OCT1 activity in some world regions. The strongest divergence in the frequency of losing OCT1 activity was observed between the populations in America and East Asia (Fig. 7a). Native Americans and East Asians, however, shared a recent common ancestor and have low genetic divergence on a genome-wide scale [37]. This suggests that a selection pressure exists either for maintaining OCT1 activity in East Asia or losing OCT1 activity in America.

Several population genetic analyses pointed to a pressure for maintaining OCT1 activity in East Asia and Oceania. First, and most important, the observed loss-of-function variability in East Asia and Oceania was significantly lower than the expected one (Tajima’s D = −1.64, *P* <0.01; Table 3). In addition, the ratio of the loss-of-function and the non-loss-of-function amino acid substitutions was more than 10-fold lower in East Asia and Oceania than in the other world regions. Second, we observed a correlation of moderate significance between loss of OCT1 activity and population migration distance (Fig. 7b). This correlation was strongly improved when East Asian populations were excluded from the analyses (Fig. 7c). In addition, significant correlation was observed between the frequencies of loss of activity of OCT1 and OATP1B1, another genetically variable drug transporter expressed in the liver (Fig. 7d) [30]. This correlation was again substantially improved when East Asian and Oceania populations were excluded from the analyses (Fig. 7e). Taken together these data suggest that a selection pressure for maintaining OCT1 activity in East Asia may be the major cause for the strong divergence in OCT1 activity between East Asia and America. Finding the cause of this pressure may shed a light on the important question about the physiological role of OCT1 in humans.

In other polymorphic genes responsible for drug metabolism and disposition, toxins have been suggested as an important source of selection pressure [38]. In the case of OCT1 selective pressure may come from food-derived organic cations or hydrophilic weak organic bases that are toxic if not rapidly detoxified in the liver. Although the truly responsible component remains unknown, we may illustrate the idea on the examples of tyramine and monocrotaline.

Tyramine is a biogenic amine that is produced by incomplete fermentation from bacteria like *Carnobacterium sp.* and *Lactobacillus sp.* [39]. Substantial amounts of tyramine and other biogenic amines are found in numerous types of bacterially fermented foods (cheese, some meat products, and red wine) [40]. Tyramine is detoxified by monoamine oxidases (MAO), which are strongly expressed in the human liver [41]. Failure to detoxify tyramine can result in increased blood pressure, migraine and nausea. Tyramine is a substrate of the human OCT1 ([29] and this work) and the alleles conferring loss of OCT1 activity are not able to transport tyramine (Fig. 4a). Therefore carriers of one and especially of zero active OCT1 alleles may experience increased plasma concentrations and suffer from adverse effects of tyramine. However, tyramine would execute a selective pressure for keeping OCT1 and cannot explain the high frequencies of loss of OCT1 activity.

A selection pressure for the loss of OCT1 activity may exist for example if loss of OCT1 activity protects the liver from toxic organic cations. An interesting example is the case of monocrotaline, a pyrrolizidine alkaloid which derives from the *Crotalaria* genus of flowering plants. Monocrotaline may be ingested as a result of contamination of food or beverages [42]. It undergoes hepatic biotransformation into severely hepatotoxic products [43], in an OCT1-dependent manner [9]. However, common *OCT1* polymorphisms result in lack of monocrotaline transport (Fig. 4a) and would thus protect the host against this toxicity. Although the global distribution of *Crotalaria* sp. that produce these types of toxins [44] does not overlap with the distribution of the loss of OCT1 activity, this substrate is proof of principle that lack of OCT1 activity could protect against ingested toxins. Furthermore, several other hepatotoxic alkaloids were recently reported to be OCT1 substrates [45, 46]. Alternatively, Oct1 deficiency in mice was reported to protect from hepatic steatosis [1] and therefore may improve fitness. However, it remains open to what extent the same effects are present in humans and it remains elusive why Native Americans would be at a higher risk of hepatic steatosis then populations in other word regions.

Alternatively, the variable frequencies of loss of OCT1 activity may result from genetic drift processes like founder effects and populations bottlenecks. This may be especially true for the high frequency of loss of OCT1 activity in Surui Indians. First, the South American Indians have traveled the longest migration distance among the Homo sapiens populations and experienced multiple founder effects [47]. This explanation is supported by the observed gradual increase in the frequency of loss of OCT1 activity from North to South America (Fig. 6). Second, Surui Indians have experienced a population bottleneck about 45 years ago when more than two-thirds of the Surui population died due to diseases introduced by the first settlers [48]. This can also explain the higher levels of the observed vs. expected nucleotide diversity in Native Americans (Tajima’s D = 1.67; Table 3).

The average non-synonymous heterozygosity in *OCT1* observed in this study (Table 3) was comparable with previous reports [16, 35]. In the context of the other organic cation transporters of the SLC22 family, the average non-synonymous heterozygosity of *OCT1* is comparable with that of *OCTN1* and substantially higher than those of *OCT2*, *OCT3*, and *OCTN2* [16, 35]. In the context of the broader group of pharmacologically-relevant membrane transporters, the average non-synonymous heterozygosity of *OCT1* is lower than the values for *VMAT1*, *CNT1*, and *PEPT2*, but is substantially higher than the values of the dopamine and serotonin reuptake transporters *DAT* and *SERT* [16]. However, simple comparisons between synonymous and non-synonymous variability have the limitation that the non-synonymous variants are automatically assumed to affect protein function. Here, after performing very broad substrate analyses, we found that five of the non-synonymous variants (26 % of the functionally tested variants) do not substantially affect OCT1 function. These variants were also among the most frequent ones, with substantial contribution to the average heterozygosity. Of the remaining variants, 12 (63 %) caused a decrease and another two caused an increase in OCT1 function. Therefore, a broader substrate-wide functional analysis of the non-synonymous polymorphisms, similar to the analyses presented here for OCT1, may be more useful for addressing the differences between functional and non-functional variability in order to get more precise information on neutrality.

One limitation of our study is the small number of individuals analyzed in some populations. For example, the observed extremely high percentage of loss of OCT1 activity in Surui Indians is based on the analysis of only eight individuals. However, even with this small number of individuals, the probability that this observation has been made by chance and that the frequency of loss of OCT1 activity in Surui Indians is similar to the frequency in Europe (the world region with the highest frequency of loss of activity besides America) or in Brahui individuals (the population with the highest frequency of loss of activity outside of America) is 6.3 × 10^−8^ or 4.8 × 10^−6^, respectively. Still, with respect to the particular case of the extremely high frequency of loss of OCT1 activity in Surui Indians, we regard our data as preliminary results that require validation.

**Fig. 8:**
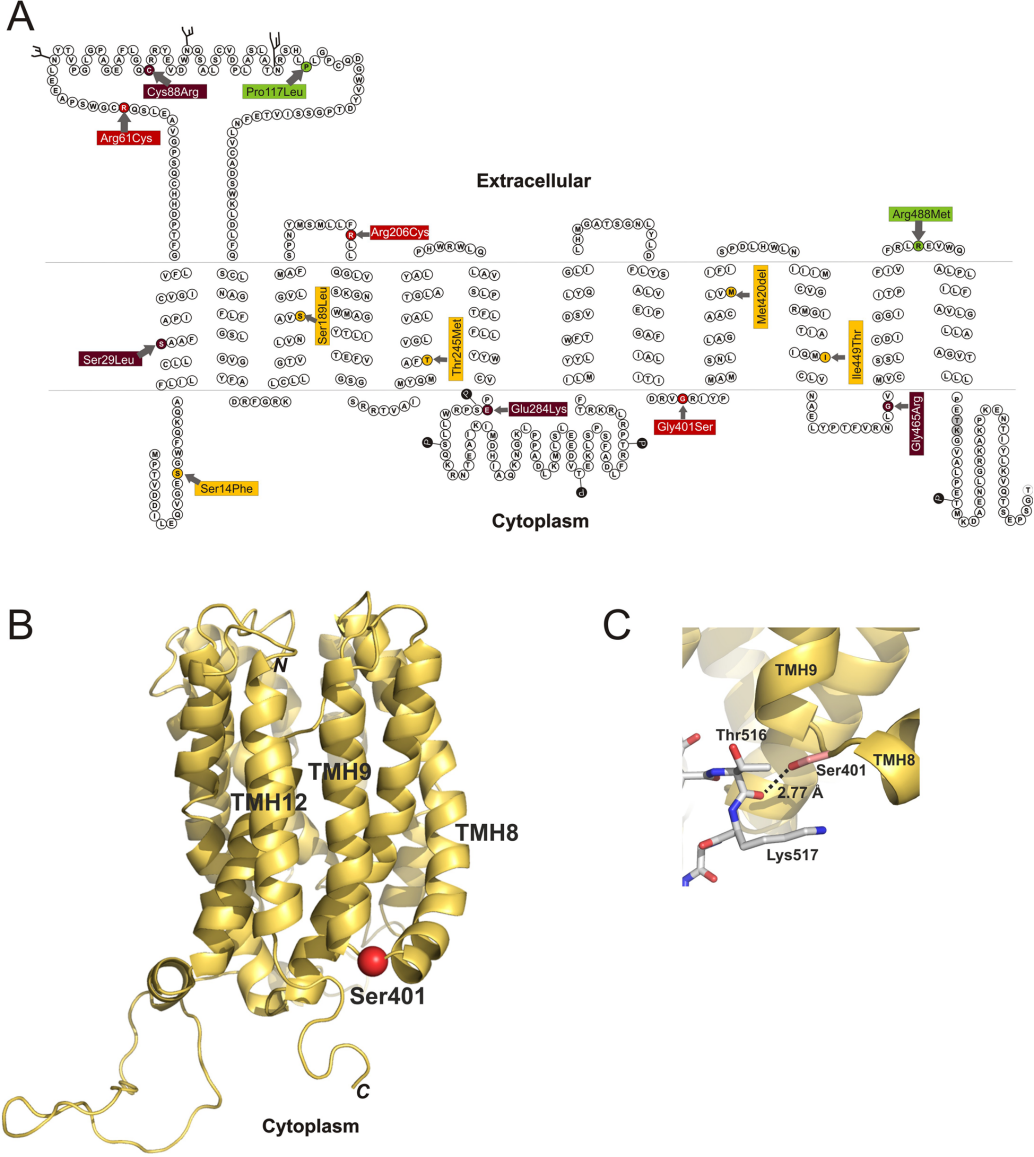
Localization of the functional amino acid substitutions within the OCT1 protein. **a** Schematic representation of the secondary structure of OCT1 with the positions of the amino acid substitutions causing strong changes in OCT1 activity indicated. Substitutions leading to substrate-specific loss of activity are shown in orange, substitutions leading to partial, but substrate-overarching, loss of activity in red, substitutions leading to complete substrate-overarching loss of activity in dark red, and substitutions leading to gain of function are shown in green. The amino acids threonine_516_ and lysine _517_, which are assumed to be involved in interaction with serine_401_ (see below), are shown in gray. Potential phosphorylation (P) and glycosylation (ψ) sites are indicated as they were predicted by Zhang *et al.* [6]. **b** A 3D model of the OCT1 protein with the position of the Gly401Ser polymorphism highlighted. Represented is a homology model of the inward-facing conformation of OCT1 (Model-ID Q9NQD4, ModBase [51]) which was visualized using PyMol software version 1.3 (Schrödinger, LLC, München, Germany). The model is based on homology with lactose permease LacY of *Escherichia coli* (PDB number 1pv6). Gly401Ser substitution is shown as a red sphere. Transmembrane helices TMH8, TMH9 and TMH12, the N terminus and C terminus are indicated. **c** A detailed representation of the region of the Gly401Ser substitution indicating a potential hydrogen bond between the hydroxyl oxygen of serine_401_ and the carbonyl oxygen of threonine_516_. A hydrogen bond could also be established between the hydroxyl oxygen of serine_401_ and the side chain amino group of lysine_517_ assuming other rotational conformations for that side chains are allowed. The position of serine_401_, threonine_516_ and lysine_517_ are shown in details. Oxygen atoms are depicted in red and nitrogen atoms in blue

Another important observation in our study is that the major reason for substrate-wide loss of OCT1 activity is improper membrane localization. With one exception (Gly401Ser), all polymorphisms that caused complete (Ser29Leu, Cys88Arg, Glu284Lys, Gly465Arg) or very strong (Arg61Cys and Arg206Cys) substrate-wide loss of OCT1 activity also cause retention of the protein in the endoplasmic reticulum. This leads to lack or a strong reduction of protein expression at the plasma membrane (Fig. 5). Several cysteine residues within the large extracellular loop between transmembrane helices one and two are involved in building intra-molecular disulfide bonds essential for the oligomerization and for the targeting of OCT1 to the plasma membrane [49]. Indeed two polymorphisms causing improper membrane localization, Cys88Arg and Arg61Cys, are located in the large extracellular loop (Fig. 8a). Cys88Arg destroys a cysteine residue known to build disulfide bonds [49] and Arg61Cys generates an alternative cysteine residue in direct proximity to an already existing one.

Not all polymorphisms that affected the membrane localization are located in the large extracellular loop. Arg206Cys is located in the second extracellular loop between transmembrane helices 3 and 4 (Fig. 8a), but it also generates an extracellular cysteine (or facing the endoplasmic reticulum during the protein synthesis). This cysteine_206_ may interact with extracellular parts of OCT1 or other molecules and act as an ‘anchor’ preventing proper folding or translocation to the plasma membrane. The other polymorphisms preventing the OCT1 localization in the cell membrane are located either in the first transmembrane domain (Ser29Leu) or in the intracellular loops (Glu284Lys and Gly465Arg). The Glu284Lys and Gly465Arg are changes in amino acids that are highly conserved between species (orthologs) and also between the members of the SLC22 family (paralogs, see Additional file 7). The members of the SLC22 family sharing this conservation recognize completely different substrates than OCT1 does. Therefore, it could be speculated that these highly conserved substrate-unspecific parts of the protein may have important structural functions and polymorphism affecting them lead to improper structure of the protein and its retention in the endoplasmic reticulum.

Gly401Ser apparently causes general impairment of the transport process without affecting the membrane localization of OCT1. Gly401Ser is located in the intracellular loop between transmembrane helices 8 and 9 (Fig. 8a). Molecular modeling suggests that this loop is in close proximity to the C-terminal end of transmembrane helix 12 and to the intracellular C-terminal tail of the protein thereafter (Fig. 8b). When the structurally flexible glycine_401_ is substituted with serine, a possible interaction may occur between the hydroxyl group of serine_401_ and the protein backbone between threonine_516_ and lysine_517_ (Fig. 8c). Accounting for the experimentally observed substrate-wide loss of OCT1 activity, we may speculate that this interaction leads to impairment of a general process like the protein plasticity essential for the transmembrane translocation of the substrate.

A substantial number of the non-synonymous polymorphisms studied showed substrate-specific loss of OCT1 activity. Five out of all 19 non-synonymous polymorphisms tested (26 %) and out of the 12 polymorphisms causing loss of OCT1 activity (42 %) showed substrate-specific effects. This number is in line with the analyses of Urban *et al.* who reported that 17 % of the non-synonymous variants in drug transporters including OCT1 show substrate-specific effects [35]. However, the number given by Urban *et al.* may be an underestimate, as it is based on the comparison of the effect of the polymorphism on only two substrates. Most polymorphisms causing substrate-specific loss of OCT1 activity are located in the transmembrane helices that are known to be involved in the formation of the binding cleft (Fig. 8) [50]. Although none of them affect amino acids known to be involved in the substrate binding [50], they do show up to 100 % reduction in the transport activity with some substrates (Fig. 4). Substrate-specific effects of the Met420del polymorphism (OCT1*2) have been reported before. No differences were observed between OCT1*2 and OCT1*1 in the uptake of MPP^+^ [11], but strong differences were observed in the uptake of metformin, tropisetron or O-desmethyltramadol [2, 4, 5]. Substrate-specific differences were also observed for the Ser14Phe and Arg61Cys polymorphisms when the uptake of metformin and vitamin B1 (which behave fairly similarly) was compared with the MPP^+^ uptake [1]. These substrate-specific effects were confirmed in the present study making artifacts from the cloning strategies or transport assays unlikely. Furthermore, our data shows that the substrate-specific effects are more general and are not restricted to the Met420del polymorphism and supports the existence of multiple binding sites that strongly vary among different OCT1 substrates.

There are important practical consequences of the high percentage of polymorphisms with substrate specific effects on OCT1 activity. It is not sufficient to test the functional effects of a newly-identified amino acid substitution using only one substrate; a variant active with one substrate may be inactive with another substrate. Thus, the concept of using model OCT1 substrates especially for predicting effects of polymorphisms on the transport activity should be critically re-evaluated. Our experiments showed only limited and strongly substrate-to-substrate variable ability of the model OCT1 substrates MPP^+^, TEA^+^, and ASP^+^ to predict the effect of polymorphisms on the uptake of the clinically relevant drugs (Additional file 12). Therefore, at least until we do have more precise information about the different substrate binding mechanisms of OCT1, the effects of polymorphisms with known substrate-specific effects of OCT1 activity should be experimentally evaluated for each new OCT1 substrate.

## Conclusions

We observed strong inter-regional variations in the frequency of loss of OCT1 activity. The differences in the frequencies of loss of OCT1 activity should be taken in consideration when transferring drug dosing recommendations from one population to another. This strong variability points the presence of an evolutionary selection for maintaining OCT1 activity in East Asian and Oceania, but the selection agent remains unknown. The major mechanistic cause of substrate-wide loss of OCT1 activity is improper membrane localization. However, a substantial number of the variants showed substrate-specific loss of activity. This reflects the poly-specificity of the OCT1 transporter and suggests different substrate binding sites and transport mechanisms for the different OCT1 substrates.

## Additional files

Additional file 1:
**Supplementary methods.**
Additional file 2:
**Validation of HEK293 cells overexpressing different OCT1 isoforms that were generated by targeted chromosomal integration.** (a) Schematic presentation of the expression plasmid pcDNA5.1 (black) integrated into the pFRT/lacZeo site of the chromosome of T-REx™ HEK293 cells (gray). The positions of the primers for validation PCRs are indicated. (b) Results of the integration-specific amplifications (PCR 1) and (PCR 2) validating the correct chromosomal integration of the overexpressing constructs. The correctness of the PCR2 sequences was validated by genotyping. (c) Quantitative real-time PCRs demonstrating strong, and homogenous among the different isoforms, expression of OCT1 in HEK293 cells. Additional file 3:
**List of the different tools used for prediction of the effects of** ***OCT1*** **polymorphisms on protein function.**
Additional file 4:
**Re-sequencing analyses of** ***OCT1.*** (a) Size of the re-sequenced regions stratified according their function: CDS, (protein) coding sequence; UTR, untranslated region, and intron-exon borders. (b) List of the 11 re-sequenced regions defined with their absolute and relative positions at chromosome 6. (c) Schematic representation of the targeted re-sequencing regions and an example of the sequencing coverage. The amplicons from the two multiplex reactions used are indicated in blue and red color. For details see the Materials and methods section. Additional file 5:
**A detailed list of genetic variants identified by massively parallel sequencing of the enriched HGDP-CEPH samples.**
Additional file 6:
***In silico*** **prediction of functional impact of amino acid substitutions observed in the worldwide genetic analyses of OCT1.**
Additional file 7:
**Evolutionary conservation of the variable OCT1 sites in OCT1 orthologs (upper part) and paralogs (lower part).**
Additional file 8:
**A detailed worldwide distribution of** ***OCT1*** **alleles including the distribution of the sub-alleles.**
Additional file 9:
**Characterization of tyramine uptake in HEK 293 cells overexpressing OCT1.** (a) Tyramine inhibits the uptake of MPP^+^. The cellular accumulation of 1-methyl-4-phenylpyridinium (MPP^+^) was measured after 1 min exposure to 10 nM ^3^H MPP^+^ in cells overexpressing OCT1*1. The graph shows mean values and standard errors of the mean from three independent experiments. (b) Concentration dependence of tyramine uptake. OCT1*1-overexpressing and control cells (stably transfected with the empty pcDNA5.1/FRT plasmid were incubated for 1 min with increasing concentrations of tyramine. The graphs show mean values and standard errors of the mean of three independent experiments. (c) The OCT1-mediated uptake. The OCT1-mediated fraction of the total uptake was calculated by subtracting the uptake in the control cells from the uptake in OCT1*1-overexpressing cells using the dataset shown in (b). Additional file 10:
**Differences in the uptake between OCT1*1 overexpressing (WT) and control cells stably transfected with the empty pcDNA5.1/FRT plasmid (pcDNA5).** The graphs show mean values and standard errors of the mean of three or more independent experiments. Additional file 11:
**Lack of interaction between Met408Val and Met420del polymorphisms in the** ***OCT1*** **gene.** (a) Comparison of the uptake activity of OCT1 carrying all theoretically possible combinations of the Met420del and Met408Val polymorphisms. The uptake was measured in HEK293 cells stably transfected with the OCT1 variants or with an empty control plasmid pcDNA5. Shown are means and standard error of the means of at least three independent experiments. (b) Subcellular localization of OCT1 isoforms carrying the four different combination of the Met420del and Met408Val polymorphisms. The protein is located on the plasma membrane indicated by co-staining with anti-Na^+^/K^+^ ATPase as a plasma membrane marker. No qualitative differences could be observed between cells carrying Met408 or Val408 independent from their background (Met420 or Del420). (c) Frequencies of the different single polymorphism genotypes (the upper part) and haplotype combinations (the lower part) in the HGDP-CEPH sample. Individual haplotypes were inferred using PHASE version 2.1. The genotype/haplotype frequencies are given as number (and %) of all individuals. Additional file 12:
**Correlation between the effects of substrate-specific loss of function OCT1 alleles on the transport of model substrates and clinically relevant drugs.** We analyzed correlations between the uptake of model OCT1 substrates (MPP^+^, TEA^+^, ASP^+^) and clinically relevant drugs (metformin and morphine) for all the *OCT1* alleles that show substrate-specific loss of activity, that is, *2, *7, *10, *11, and *13. Shown are means and standard error of the means of at least three independent transport experiments. Solid lines represent linear regression, dashed lines represent an optimal theoretical correlation with identical effects of the loss-of-function allele on both the model substrates and drug. * denotes significant correlations with *P* <0.05. Additional file 13:
**Correct subcellular localization of** ***OCT1*** **alleles *1, *2, *8, *9, *10, *11, and *13.** The analyzed alleles are known to have normal (*OCT1*1*, **1B*, **1C*, and **1D*), increased (*OCT1*8* and **9*) or substrate-specific loss of OCT1 activity (*2, *10, *11, and *13). The subcellular localization of the different OCT1 isoforms was analyzed after immunocytochemical staining of OCT1 (green) in combination with Na^+^/K^+^ ATPase (red, upper part) as a marker for plasma membrane and calnexin (red, lower part) as a marker for endoplasmic reticulum. Additional file 14:
**Detailed maps of the interpopulation variation of the frequency of loss of OCT1 activity in Europe (a) and China (b).**
Additional file 15:
**Pairwise analyses of divergence based on the frequencies of OCT1 alleles in populations and in different world regions (the upper panel).** The deep blue color correspond to high divergence between the populations / regions (high F_ST_ values). We analyzed 62 population from the 1000 Genomes Project (designated with 1 K) and the HGDP-CEPH project (the rest). The populations were stratified in world regions as listed in Table 1. In contrast to Fig. 7, which shows divergence at the level of loss of OCT1 activity, this figure shows divergences at the level of single OCT1 alleles.
