# Supplementary material for: Global genetic analyses reveal strong inter-ethnic variability in the loss of activity of the organic cation transporter OCT1
Source: Genome Med. 2015 Jun 18;7(1):56. doi: 10.1186/s13073-015-0172-0 (PMC4495841; doi:10.1186/s13073-015-0172-0)
Supplement: Additional file 1: — Supplementary methods. [file 13073_2015_172_MOESM1_ESM.docx]

**Supplementary methods Seitz, Stalmann *et al.***

##### Massively parallel re-sequencing

Eleven regions covering the eleven *OCT1* exons and their flanking regions were enriched by PCR and re-sequenced using semiconductor-based massively parallel sequencing on the Ion Torrent PGM (Life Technologies, Darmstadt, Germany). The enrichment was carried out in two parallel multiplex PCRs. The first multiplex PCR contained 9 amplicons 221 to 336 bp in length. The PCR reaction was carried out using the QIAGEN Multiplex PCR Kit (Qiagen, Hilden, Germany) under the following conditions: initial denaturation at 95^o^C for 15 min, 35 cycles of 94^o^C for 30 sec, 64.8^o^C for 90 sec and 72^o^C for 60 sec, and a final elongation at 72^o^C for 10 min. The second multiplex PCR reaction contained 7 amplicons 198 to 254 bp in length. The PCR reaction was carried out using the QIAGEN multiplex PCR kit under the following conditions: initial denaturation at 95^o^C for 15 min, 35 cycles of 94^o^C for 30 sec, 58.1^o^C for 90 sec and 72^o^C for 60 sec, and a final elongation at 72^o^C for 10 min. The primer concentrations were optimized to assure similar molecular ratios of the different products (primer sequences and concentrations are given in supplementary table S1). The first and second multiplex reactions were quantified by qPCR and pooled together in equimolar ratios. The samples (including the repetitions) were grouped in 52 pools of up to 24 samples each and subjected to library preparation for sequencing. The library preparation was carried out with the IonXpress^TM^ Plus Fragment Library Kit (Life Technologies) using 1-16 IonXpress^TM^ barcoded adaptor set according to the manufacturer’s instructions. The library was quantified using the GeneRead library quantification kit (Qiagen) and a total of 3x10^7^ molecules from 16 libraries, labelled with different molecular barcodes, were pooled together and used for template preparation. The template preparation was carried out with Ion OneTouch^TM^ using the Ion OneTouch ^TM^ 200 Template Kit according to the manufacturer’s instructions. The template-containing Ion Sphere^TM^ particles were enriched using the E/S module (Life Technologies) and sequenced on Ion PGM (Life Technologies) on 316^TM^ chips using 200 bp mode (520 run flows). Data analyses were performed with Torrent Suite v3.6.2 and genetic variants were called using variantCaller v 3.6.63335 (Life Technologies).

##### Genotyping of selected SNPs

Sixteen genetic variants were genotyped in two single-base primer-extension reactions with florescence detection (SNaPhot®, Life Technologies): Ser14Phe (rs34447885), Ser29Leu (rs375175439), Arg61Cys (rs12208357), Cys88Arg (rs55918055), Gln97Lys, Pro117Leu (rs200684404), Ser189Leu (rs34104736), Arg206Cys, Gly220Val (rs36103319), Thr245Met, Glu284Lys, Gly401Ser (rs34130495), Gly414Ala (rs72552762), Met420del (rs202220802), Ile449Thr (rs183240019), aGly465Arg (rs34059508), and Arg488Me(rs35270274). In the first reaction, four regions containing the variants Ser14Phe, Arg61Cys, Cys88Arg, Gln97Lys, Pro117Leu, Ser189Leu, Arg206Cys, Gly220Val, Gly401Ser, Met420del, and Gly465Arg were pre-amplified by multiplex PCR using the QIAGEN Multiplex PCR Kit (Qiagen) under the following conditions: initial denaturation at 95^o^C for 15 min, 35 cycles of 94^o^C for 30 sec, 65^o^C for 90 sec and 72^o^C for 60 sec, and a final elongation at 72^o^C for 10 min. In the second reaction, five regions containing the variants Ser29Leu, Thr245Met, Glu284Lys, Gly414Ala, Ile449Thr and Arg488Met were pre-amplified by multiplex PCR using the QIAGEN Multiplex PCR Kit (Qiagen) under the following conditions: initial denaturation at 95^o^C for 15 min, 35 cycles of 94^o^C for 30 sec, 58^o^C for 90 sec and 72^o^C for 60 sec, and a final elongation at 72^o^C for 10 min. The PCR products were purified by incubation for one hour at 37^o^C with 2 U shrimp alkaline phosphatase (USB/Affymetrix, Staufen, Germany) and 3 U exonuclease I (*E. coli*, MBI Fermentas, Thermo Fischer, St. Leon-Rot, Germany). The primer extension reaction was carried out using the SNaPshot® Kit (Life Technology) according the manufacturer’s instructions. The reaction products were analyzed using the 3130xl Genetic Analyzer and the genotypes were called using GeneMapper® v3.7 (both from Life Technology). Primer sequences and their final concentrations in the pre-amplification PCR and primer-extension reactions are given in supplementary table S2.

Five variants were genotyped by real-time PCR. The variants Phe160Leu (rs683369), Pro341Leu (rs2282143), Arg342His (rs34205214), and Met408Val (rs628031) were genotyped with the pre-designed TaqMan® SNP genotyping assays C____928536_30, C__15877554_40, C__30634086_10, and C___8709275_60, respectively. The variant Arg488Met (rs35270274) was genotyped using the customer TaqMan® SNP genotyping assay with the primers 5’-GGG ATA ATC ACC CCC TTC ATA GTC T-3’ and 5’-CAC TCA GTT CCA CCC ATG CT-3’ and probes VIC-AGG CTG AGG GAG GTC-MGB and FAM-AGG CTG ATG GAG GTC-MGB. All real-time PCR genotyping assays were obtained by Life Technologies and were run under standard manufacturer’s instructions.

Sex matching was performed to exclude sample swaps [1]. Five percent of the samples were genotyped in duplicate, showing 100% concordance in the genotype calls.

##### Construction and characterization of OCT1 overexpressing cell lines

The OCT1-overexpressing HEK293 cells were generated by targeted chromosomal integration using the Flp-In system^TM^ (Life Technologies, Darmstadt, Germany). The construction and characterization of the cells overexpressing OCT1 alleles *1 (wild type), *3, *4, *5 and *6 were described in detail before [2]. The cells overexpressing the remaining OCT1 alleles were constructed following the same procedure. Briefly, point mutations leading to the amino acid substitutions analyzed were introduced by targeted mutagenesis in a pcDNA3::OCT1 plasmid (kindly supplied by Hermann Koepsell and Valentin Gorboulev, University of Würzburg, Germany). The primers used are listed in supplementary table 3. The complete *OCT1* open reading frame was sequenced to confirm the presence of the required mutations and the correctness of the rest of the gene. *OCT1* was recloned into the pcDNA5.1 vector (Life Technologies, Darmstadt, Germany) using *HindIII* and *EcoRV* restriction sites. The resulting plasmids were integrated into the genome of T-Rex^TM^ HEK293 cells using the Flp-In system^TM^ (Life Technologies, Darmstadt, Germany) according to the manufacturer’s instructions. Clones carrying the stable transfection were selected by the addition of 300 µg/ml of Hygromycin B to the media. After the selection of single clones, the concentration of Hygromycin B was reduced to 100 µg/ml. The correct chromosomal integration was confirmed by integration-specific PCR [2] and the overexpression of *OCT1* variants was confirmed by RT-qPCR (Supplementary fig. S1).

##### OCT1 activity assays

The OCT1-mediated uptake of tropisetron and debrisoquine was measured as described before [2, 3]. Briefly, 9 million cells were plated on 100 mm Petri dishes (BD Falcon, Heidelberg, Germany) that were pre-coated with poly-D-lysine (1-4 kDa, Sigma-Aldrich, Taufkirchen, Germany). After 48 hours the cells were washed once with 10 ml 37°C-warm Hank’s buffered salt solution (HBSS; Invitrogen, Karlsruhe, Germany). Uptake measurements were performed by adding 5 ml 37°C-warm HBSS containing tropisetron or debrisoquine for 3 and 1 min, respectively. All uptake measurements were performed at pH of 7.4. The uptake was stopped by adding 20 ml ice cold HBSS. The cells were washed with 20 ml ice cold HBSS, and 2 ml of ice cold HBSS were added. The cells were detached from the surface with the help of a cell scraper and transferred into a 2 ml reaction tube. The cells were lysed in 80% acetonitrile and 20% 50 mM sodium acetate (pH 5). The intracellular amount of tropisetron and debrisoquine were quantified by HPLC as described before [2, 3].

The uptake of all other substances was measured in 12 well plates. First, 6x10^5^ cells were plated per well of poly-D-Lysine pre-coated 12-well plates (Nunc, Langenselbold, Germany). The cells were grown for 48 h to reach confluence and washed once with 2 ml 37°C-warm HBSS. The uptake reaction was started by adding 400 µl 37°C-warm HBSS containing the substrate and the cells were incubated for 1 or 2 min depending on the substrate used. The uptake was stopped by adding 2 ml ice cold HBSS. The cells were washed twice with 2 ml ice cold HBSS. The intracellular concentration of MPP^+^, TEA^+^, morphine, metformin and tyramine were quantified by radioactive detection. The cells were lysed in 500 µl 0.1 N NaOH containing 0.1% SDS. Four hundred µl of the lysates were mixed with 9 ml Aquasafe 500+ liquid scintillator (Zinsser Analytics, Frankfurt am Main, Germany) and the intracellularly accumulated substrate was quantified in a liquid scintillation counter (Beckman LS5000TD from Beckman Coulter, Krefeld, Germany). The intracellular ASP^+^ was quantified using fluorescence detection. The cells were lysed in 500 µl RIPA buffer (50 mM Tris-HCl pH 7.6, 150 mM NaCl, 1 mM EDTA, 1% NP-40, 0.25% Na-deoxycholate and 0.1% SDS) and fluorescence was measured at wavelengths of 485 nm excitation and 612 nm emission. The intracellular O-desmethyltramadol and monocrotaline concentrations were quantified by LC-MS/MS. The cells were lysed with 500 µl 80 % acetonitrile and the cell debris were removed by centrifugation at 16500 g for 15 min. Three hundred µl of the supernatant were transferred to a 10 ml glass tube and evaporated at 40 ^o^C under nitrogen flow. The residue was re-constituted in 150 µl mobile phase (0.1% (v/v) formic acid, 12.85% Acetonitrile und 2.17% Methanol) and 10 µl were used for the quantification. The quantification was performed on an ABI 4000 LC-MS/MS system (Life Technologies, Darmstadt, Germany) using a Brownlee SPP RP-Amide Column (4.6x100 mm, 2.7 µm; PerkinElmer, Waltham, MA, USA), with a SecurityGuard C18, 4*2mm pre-column (Phenomenex, Aschaffenburg, Germany). O-desmethyltramadol and monocrotaline were detected using multiple reaction monitoring (MRM) mode of transition at m/z of 250.2 > 58.1 (retention time 4.2 min) and 326.2 > 120.2 (retention time 3.3 min), respectively. Atropine was used as internal standard and was detected at m/z of 290.2 > 142.2 (retention time 5.1 min).

In all transport measurements, the intracellular amount of the drug was normalized to the total protein of the sample as measured using the bicinchoninic acid assay [4].

##### Western blot analysis

Approximately two million cells were lysed for 10 min in 300 µl ice-cold RIPA buffer supplemented with 1 mM phenylmethanesulfonylfluoride (PMSF) and protease inhibitors (Halt^TM^; Pierce, Rockford, USA). The cell debris were removed by centrifugation (10 min at 13.000 rpm, 4 °C) and the total cellular protein in the supernatant was quantified using a bicinchoninic acid assay [4].

Ten to thirteen µg total cellular extracts were separated on a 12% SDS gel and transferred to a polyvinylidene difluoride membrane using semi-dry blotting. The membrane was blocked for 1 h at room temperature using TBST (20 mM Tris-HCl pH 7.6, 150 mM NaCl, 0.05% Tween 20) supplemented with 5% non-fat dry milk. The primary antibodies were diluted in TBST supplemented with 5% non-fat dry milk, and added to the membrane and incubated overnight at 4 °C. As primary antibodies we used mouse monoclonal anti-OCT1 (2C5; Novus Biologicals, Cambridge, UK) diluted 1:400 or mouse monoclonal anti-GAPDH (6C5; Zytomed, Berlin, Germany) diluted 1:50,000. The membranes were washed with TBST three times for 5 min at room temperature, incubated for 1 h with the secondary antibody in TBST with 0.5% non-fat dry milk, washed again with TBST three times for 5 min and finally washed with Tris-buffered saline. As a secondary antibody we used horseradish peroxidase-conjugated anti-mouse antibody (BioMol, Hamburg, Germany) diluted 1:10,000. Horseradish peroxidase activity was detected by chemiluminescence using SuperSignal® West Pico (for detecting GAPDH) or SuperSignal® West Femto Kits (for detecting OCT1; Thermo Scientific, Waltham, USA) according to the manufacturer’s instructions. The VersaDoc^TM^ imaging system with Quantity One software v4.6.7 (BioRad, München, Germany) was used to visualize the signal.

The protein deglycosylation was carried out as follows: 22 µg total protein extracts were denatured by incubation for 5 min at 95 °C in buffer containing 60 mM Tris HCl pH 6.8, 0,002% bromophenol blue, 100 mM dithiothreitol, 7% glycerol, and 2% sodium dodecyl sulfate. For deglycosylation with Endoglycosidase H, 1250 units Endoglycosidase H (*Endo*H, New England Biolabs, Frankfurt am Main, Germany) and sodium citrate to a final concentration of 50 mM were added and the reaction was incubated for 1 hour at 37°C. For deglycosylation with N-Glycosidase F, 500 units N-Glycosidase F (*PNGase*F, New England Biolabs), NP-40 and sodium citrate were added to a final concentration of 1% and 50 mM, respectively. The reaction was incubated for 1 hour at 37°C. The deglycosylated samples were directly applied to a SDS-PAGE as described above.

**Supplementary tables**

**Table S1. Sequences and concentrations of the primers used in the massively parallel resequencing reaction.**

|  | **Primer ID** | **Primer length [bp]** | **Sequence** | **Final concentration [µM]** | **Amplicon size [bp]** |
| --- | --- | --- | --- | --- | --- |
| **Multiplex PCR 1** | Exon1_f4 | 24 | TGGCCACGTGCATTCTTCCTTTTC | 0.2 | 242 |
|  | Exon1_r4 | 24 | CTCCCCAACCTGCTCCAGAATGTC | 0.2 |  |
|  | Exon1_f2c | 23 | TGGGCATCGTCTTCCTGGGTTTC | 0.12 | 241 |
|  | Exon1_r2c | 22 | CGGCAGGTGGCTCCTGTTGGTG | 0.12 |  |
|  | Exon3_f1 | 23 | TGACCCCAGATTTCAACCTCTCC | 0.2 | 235 |
|  | Exon3_r1 | 23 | CCTGGCCTCATCCCCATGATAAT | 0.2 |  |
|  | Exon4_f1 | 25 | GGGCTCCTGGGCTCCTGCAAGGAAC | 0.15 | 257 |
|  | Exon4_r1 | 23 | CGCCCCTTCCCCGTAGGCAGGAG | 0.15 |  |
|  | Exon6_f1 | 25 | TCCCAGGTGGCTCTGCTCATGACAG | 0.1 | 221 |
|  | Exon6_r1 | 25 | GTGCTTGGGCGAGCGTGCTGATTCT | 0.1 |  |
|  | Exon7_f1e | 22 | CGGGAACCTCTACCTGGATTTC | 0.8 | 247 |
|  | Exon7_r1e | 25 | TTCCAGAGGCTTATCAAAGAGTCAC | 0.8 |  |
|  | Exon8_f1 | 25 | ACCCACCATGGCCTCTCACAGTAAC | 0.12 | 336 |
|  | Exon8_r1 | 24 | GGTGGGTTCACAGCCATTTCACTG | 0.12 |  |
|  | Exon10_f1 | 25 | TTGGCTGGCTGTGATTATTTCTGTA | 0.2 | 233 |
|  | Exon10_r1 | 23 | TTTGGAGGGCGTGTCAGACTCAT | 0.2 |  |
|  | Exon11_f2b* | 22 | GGCACCTGAGAGAGATGTTTTG | 0.5 | 228 |
|  | Exon11_r2b* | 24 | ATCGAATGCACAGGTGGAAGATAG | 0.5 |  |
| **Multiplex PCR 2** | Exon1_f1c | 25 | CGGCCATGAGCATGCTGAGCCATCA | 0.12 | 198 |
|  | Exon1_r1d | 25 | TCAGCTCAGCCACCCCAGGACTCTG | 0.12 |  |
|  | Exon1_f3 | 24 | AGCCCTGCGGAGGAGCTGAACTATA | 0.3 | 254 |
|  | Exon1_r3 | 25 | GTCCCAGGAACTCCCATGTTACAGA | 0.3 |  |
|  | Exon2_f1* | 25 | ATGGAAGGGTGTAGTCCTGACTCAC | 0.1 | 239 |
|  | Exon2_r1* | 25 | CGCCCTTAGAATCTGTTCTCATGAC | 0.1 |  |
|  | Exon5_f1 | 25 | CCCCGCTCAGGGCTGAACGTCAGAC | 0.2 | 254 |
|  | Exon5_r1b | 25 | CTGGCAGCCCCAGACGAATCTGCAC | 0.2 |  |
|  | Exon7_f2b | 26 | AGGGCATTCTAAACCCAGTGATTCAT | 0.15 | 222 |
|  | Exon7_r2b | 20 | CCCGGGATTTCGACCAGAGC | 0.15 |  |
|  | Exon9_f1c | 25 | TGGAAAAGTGAATCACAGAATTATC | 0.6 | 206 |
|  | Exon9_r1c | 25 | GCTGCAAAAGAAGGAATGATATATG | 0.6 |  |
|  | Exon11_f1 | 25 | TTTGCAACAGTTCCATCATCAACAA | 0.7 | 202 |
|  | Exon11_r1 | 25 | TGCAGAGGATAACTCCATCTTCATC | 0.7 |  |

* PCR reactions used for quantification of the multiplex products before pooling (for details see the text)

**Table S2. Sequences and concentrations of the primers used for single variant genotyping.**

| **Reaction** | **Reaction type** | **Primer ID** | **Primer sequences* (5'->3')** | **Final concentra-tion [µM]** |
| --- | --- | --- | --- | --- |
| Reaction 1 | pre-amplification PCR | OCT1_1_for | CATGCCCACCGTGGATGACATTCTG | 0.2 |
|  |  | OCT1_1_rev | CTGGCCCCCGACTTGTGCTAGCTGT | 0.2 |
|  |  | OCT1_3_for | GCGCCACCAGCGGGAACCTCTACCT | 0.3 |
|  |  | OCT1_3_rev | AACCTCCGCCTCCTGGGTTCAAGCA | 0.3 |
|  |  | OCT1_4_for | TCTGGGCTGGTCCTCATGGTTCCTC | 0.2 |
|  |  | OCT1_4_rev | GCCAGACCTCCCTCAGCCTGAAGAC | 0.2 |
|  |  | OCT1_5_for | TGGCATCCCACCATGCATGTCTGAC | 0.2 |
|  |  | OCT1_5_rev | ACTGGTGCCCCGCAAGCTCCTTGAC | 0.2 |
|  | SNaPshot | Ser14Phe | gaTCTGGAGCAGGTTGGGGAGT | 0.05 |
|  |  | Ser189Leu | gagatcAGAAGGCCATGAGCACGCCC | 0.05 |
|  |  | Arg61Cys | gatcgatcTGGGGTGGCTGAGCTGAGCCAG | 0.1 |
|  |  | Gln97Lys | gatcgatcgatcgTACACAGCTGAGGGCGCTCT | 0.05 |
|  |  | Pro117Leu | gatcgatcgatcgatcgCACCAACAGGAGCCACCTGC | 0.1 |
|  |  | Arg206Cys | gatcgatcgatcgatcgatcGACCAGGCCCTGCAGCAGGC | 0.05 |
|  |  | Gly401Ser | gatcgatcgatcgatcgatcCATGGCCATGGGGTAGATGCGGC | 0.1 |
|  |  | Met420del | gatcgatcgatcgatcgatcgatcgatGCGGGGGCAGCCTGCCTCGTCAT | 0.15 |
|  |  | Gly465Arg | gatcgatcgatcgatcgatcgatcgatcgatcAGGGAGGAACACACCATCACTC | 0.15 |
|  |  | Cys88Arg | gatcgatcgatcgatcgatcgatcgatcgatcgatcgaGTCCACTTCATAGCGCCTGC | 0.15 |
|  |  | Gly220Val | gatcgatcgatcgatcgatcgatcgatcgatcgatcgatcgatCAAGGGCAACTGGATGGCTG | 0.25 |
| Reaction 2 | pre-amplification PCR | Exon 1_for | CGGCCATGAGCATGCTGAGCCATCA | 0.3 |
|  |  | Exon 1_rev | GTCCCAGGAACTCCC**R**TGTTACAGA | 0.3 |
|  |  | Exon 4_for | GGGCTCCTGGGCTCCTGCAAGGAAC | 0.2 |
|  |  | Exon 4_rev | CGCCCCTTCCCCGTAGGCAGGAG | 0.2 |
|  |  | Exon 5_for | CCCCGCTCAGGGCTGAACGTCAGAC | 0.2 |
|  |  | Exon 5_rev | CTGGCAGCCCCAGA**Y**GAATCTGCAC | 0.2 |
|  |  | Exon 7_for | AGGGCATTCTAAACCCAGTGATTCAT | 0.3 |
|  |  | Exon 7_rev | TTCCAGAGGCTTATCAAAGAGTCAC | 0.3 |
|  |  | Exon 8_for | ACCCACCATGGCCTCTCACAGTAAC | 0.2 |
|  |  | Exon 8_rev | GGTGGGTTCACAGCCATTTCACTG | 0.2 |
|  | SNaPshot | Ser29Leu | gagatcAGATGGGCGCAAAGGCAGCC | 0.2 |
|  |  | Glu284Lys | gatcgatcgaTAACAGCCACC**R**AGGGGACT | 0.2 |
|  |  | Thr245Met | gatcgatcgatcgTAAGCGCCACCAGCCCCACC | 0.2 |
|  |  | Gly414Ala | gatcgatATGGCCRTGTCAAATTTGTTGGCRG | 0.6 |
|  |  | Ile449Thr | gatcgatcgatcgatcgatcGCTCAGCATTCACCAGGCAG | 0.6 |
|  |  | Arg488Met | gatcgatcgatcgatcgatcgatCTTCATAGTCTTCAGGCTGA | 0.2 |
|  |  |  |  |  |

* Lowercase letters denote the sequence-unspecific part of the SNaPshot^TM^ primer. Degenerate bases used at positions known to be polymorphic are highlighted in bold.

**Table S3. Sequence of the primers used in site-directed mutagenesis.**

| **Mutation** | **Primer sequence* (5'->3')** |
| --- | --- |
| Ser14Phe_for | TCTGGAGCAGGTTGGGGAGT**T**TGGCTGGTTCCAGAAGCAAG |
| Ser14Phe_rev | CTTGCTTCTGGAACCAGCCA**A**ACTCCCCAACCTGCTCCAGA |
| Ser29Leu_for | ATCTTATGCCTGCTGT**T**GGCTGCCTTTGCGCCC |
| Ser29Leu_rev | GGGCGCAAAGGCAGCC**A**ACAGCAGGCATAAGAT |
| Pro117Leu_for | CACCAACAGGAGCCACCTGC**T**GCTGGGTCCCTGCCAGGATG |
| Pro117Leu_rev | CATCCTGGCAGGGACCCAGC**A**GCAGGTGGCTCCTGTTGGTG |
| Phe160Leu_for | TGAATGCGGGCTTCTT**G**TTTGGCTCTCTCGGTG |
| Phe160Leu_rev | CACCGAGAGAGCCAAA**C**AAGAAGCCCGCATTCA |
| Ser189Leu_for | TGTGCTGGTCAACGCGGTGT**T**GGGCGTGCTCATGGCCTTCT |
| Ser189Leu_rev | AGAAGGCCATGAGCACGCCC**A**ACACCGCGTTGACCAGCACA |
| Arg206Cys_for | ACATGTCCATGCTGCTCTTC**T**GCCTGCTGCAGGGCCTGGTC |
| Arg206Cys_rev | GACCAGGCCCTGCAGCAGGC**A**GAAGAGCAGCATGGACATGT |
| Thr245Met_for | TACCAGATGGCCTTCA**T**GGTGGGGCTGGTGGCG |
| Thr245Met_rev | CGCCACCAGCCCCACC**A**TGAAGGCCATCTGGTA |
| Glu284Lys_for | CTACTGGTGTGTGCCG**A**AGTCCCCTCGGTGGCT |
| Glu284Lys_rev | AGCCACCGAGGGGACT**T**CGGCACACACCAGTAG |
| Pro341Leu_for | GACCTGTTCCGCACGC**T**GCGCCTGAGGAAGCGC |
| Pro341Leu_rev | GCGCTTCCTCAGGCGC**A**GCGTGCGGAACAGGTC |
| Arg342His_for | CCTGTTCCGCACGCCGC**A**CCTGAGGAAGCGCACCT |
| Arg342His_for | AGGTGCGCTTCCTCAGG**T**GCGGCGTGCGGAACAGG |
| Gly414Ala_for | TCAAATTTGTTGGCGG**C**GGCAGCCTGCCTCGTC |
| Gly414Ala_rev | GACGAGGCAGGCTGCC**G**CCGCCAACAAATTTGA |
| Ile449Thr_for | ATTGCAATACAAATGA**C**CTGCCTGGTGAATGCT |
| Ile449Thr_rev | AGCATTCACCAGGCAG**G**TCATTTGTATTGCAAT |
| Met408Val_for | CATCTACCCCATGGCC**G**TGTCAAATTTGTTGGC |
| Met408Val_rev | GCCAACAAATTTGACA**C**GGCCATGGGGTAGATG |
| Met420del_for | GGGCAGCCTGCCTCGTCATTTTTATCTCACCTGA |
| Met420del_rev | TCAGGTGAGATAAAAATGACGAGGCAGGCTGCCC |
| Arg488Met_for | ATAGTCTTCAGGCTGA**T**GGAGGTCTGGCAAGCCT |
| Arg488Met_rev | AGGCTTGCCAGACCTC**C**ATCAGCCTGAAGACTAT |

* Mutated bases are highlighted in bold. The affected codons are underlined.

**References**

1. Tzvetkov MV, Meineke I, Sehrt D, Vormfelde SV, Brockmoller J: **Amelogenin-based sex identification as a strategy to control the identity of DNA samples in genetic association studies.** *Pharmacogenomics* 2010, **11:**449-457.

2. Saadatmand AR, Tadjerpisheh S, Brockmoller J, Tzvetkov MV: **The prototypic pharmacogenetic drug debrisoquine is a substrate of the genetically polymorphic organic cation transporter OCT1.** *Biochem Pharmacol* 2012, **83:**1427-1434.

3. Tzvetkov MV, Saadatmand AR, Bokelmann K, Meineke I, Kaiser R, Brockmoller J: **Effects of OCT1 polymorphisms on the cellular uptake, plasma concentrations and efficacy of the 5-HT(3) antagonists tropisetron and ondansetron.** *Pharmacogenomics J* 2012, **12:**22-29.

4. Smith PK, Krohn RI, Hermanson GT, Mallia AK, Gartner FH, Provenzano MD, Fujimoto EK, Goeke NM, Olson BJ, Klenk DC: **Measurement of protein using bicinchoninic acid.** *Anal Biochem* 1985, **150:**76-85.
