# Supplementary figures and images for: Global genetic analyses reveal strong inter-ethnic variability in the loss of activity of the organic cation transporter OCT1
Source: Genome Med. 2015 Jun 18;7(1):56. doi: 10.1186/s13073-015-0172-0 (PMC4495841; doi:10.1186/s13073-015-0172-0)

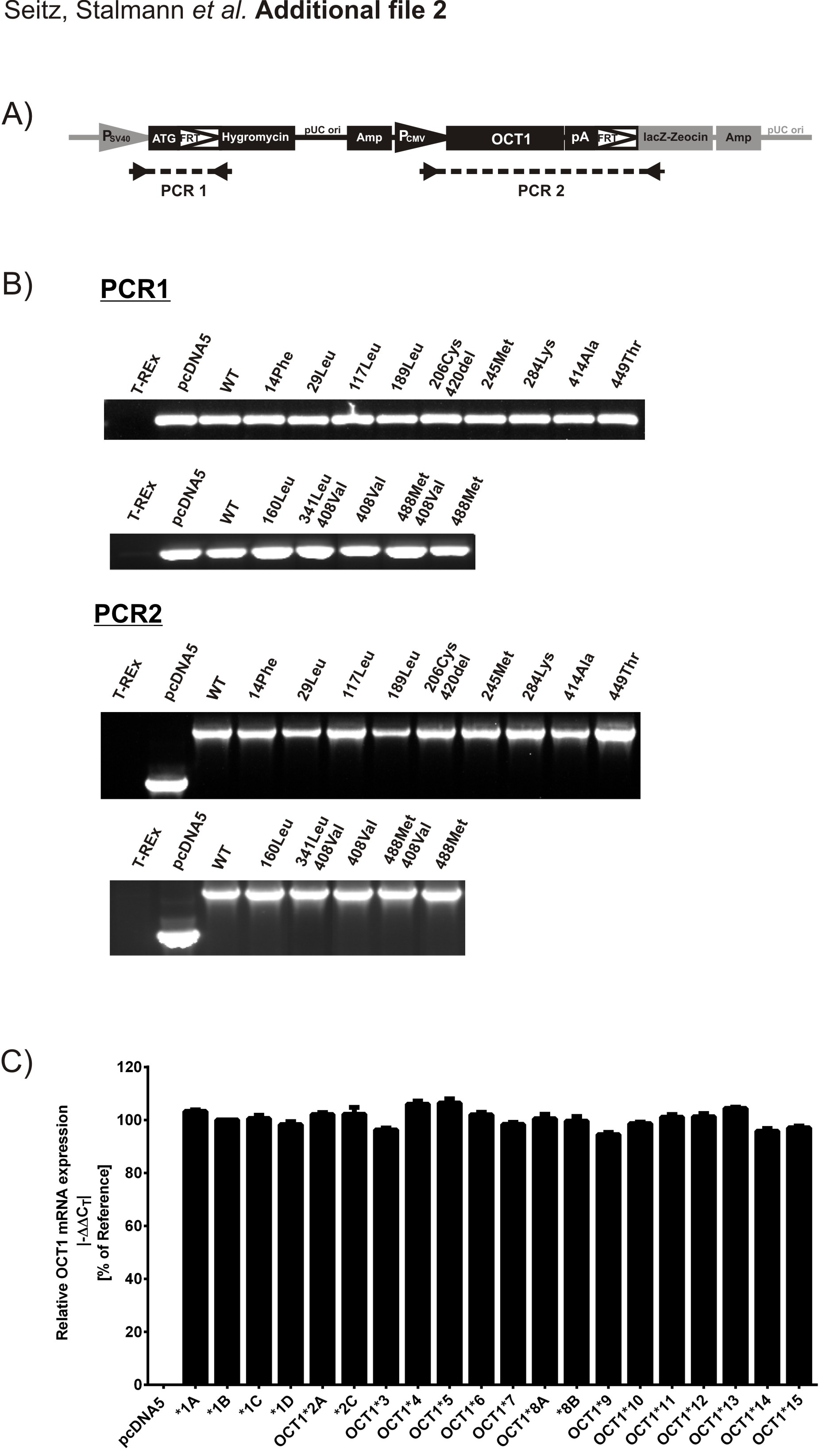

Supplement: Additional file 2: — Validation of HEK293 cells overexpressing different OCT1 isoforms that were generated by targeted chromosomal integration. (a) Schematic presentation of the expression plasmid pcDNA5.1 (black) integrated into the pFRT/lacZeo site of the chromosome of T-REx™ HEK293 cells (gray). The positions of the primers for validation PCRs are indicated. (b) Results of the integration-specific amplifications (PCR 1) and (PCR 2) validating the correct chromosomal integration of the overexpressing constructs. The correctness of the PCR2 sequences was validated by genotyping. (c) Quantitative real-time PCRs demonstrating strong, and homogenous among the different isoforms, expression of OCT1 in HEK293 cells. [file 13073_2015_172_MOESM2_ESM.jpg]

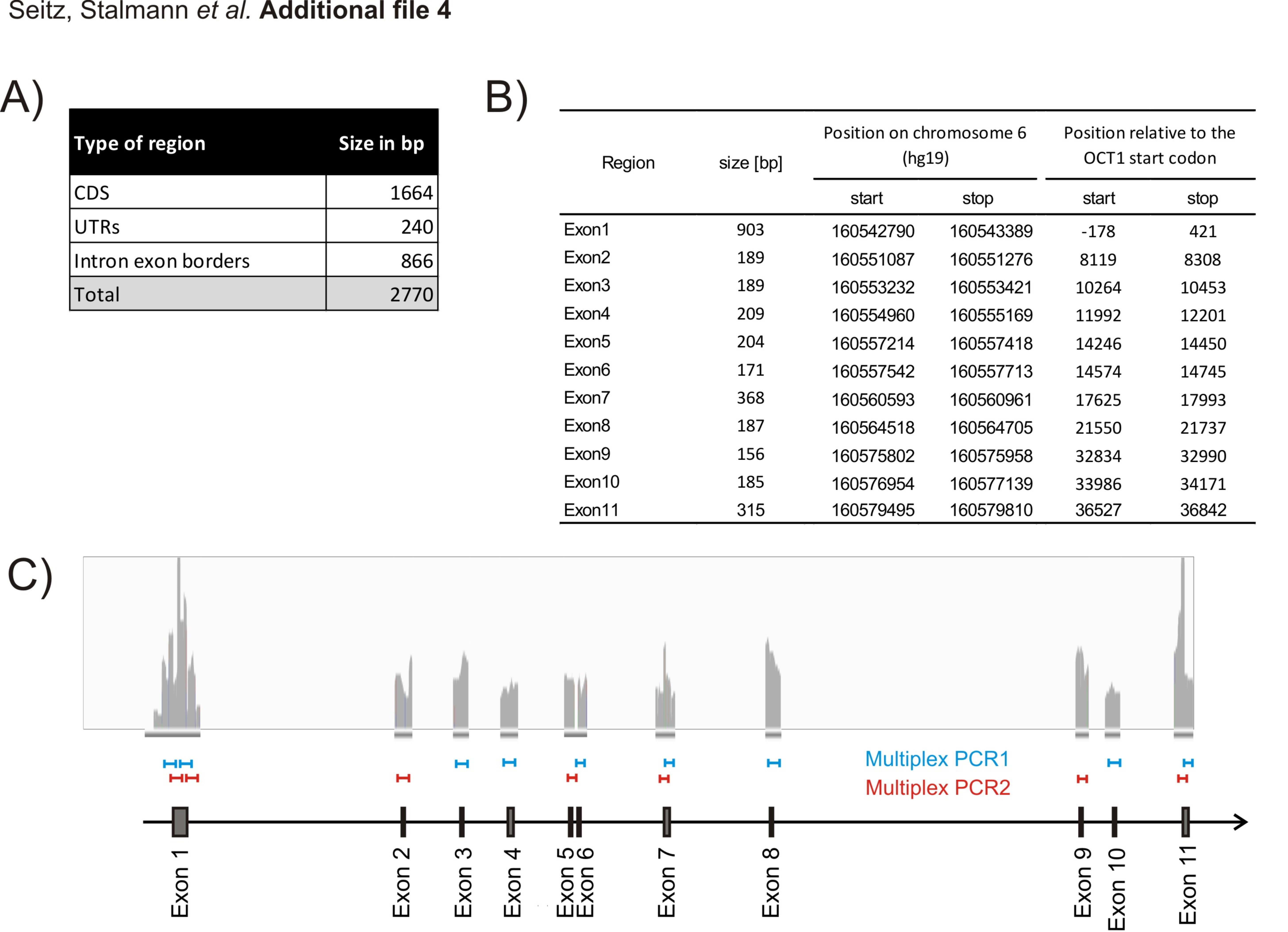

Supplement: Additional file 4: — Re-sequencing analyses of OCT1. (a) Size of the re-sequenced regions stratified according their function: CDS, (protein) coding sequence; UTR, untranslated region, and intron-exon borders. (b) List of the 11 re-sequenced regions defined with their absolute and relative positions at chromosome 6. (c) Schematic representation of the targeted re-sequencing regions and an example of the sequencing coverage. The amplicons from the two multiplex reactions used are indicated in blue and red color. For details see the Materials and methods section. [file 13073_2015_172_MOESM4_ESM.jpg]

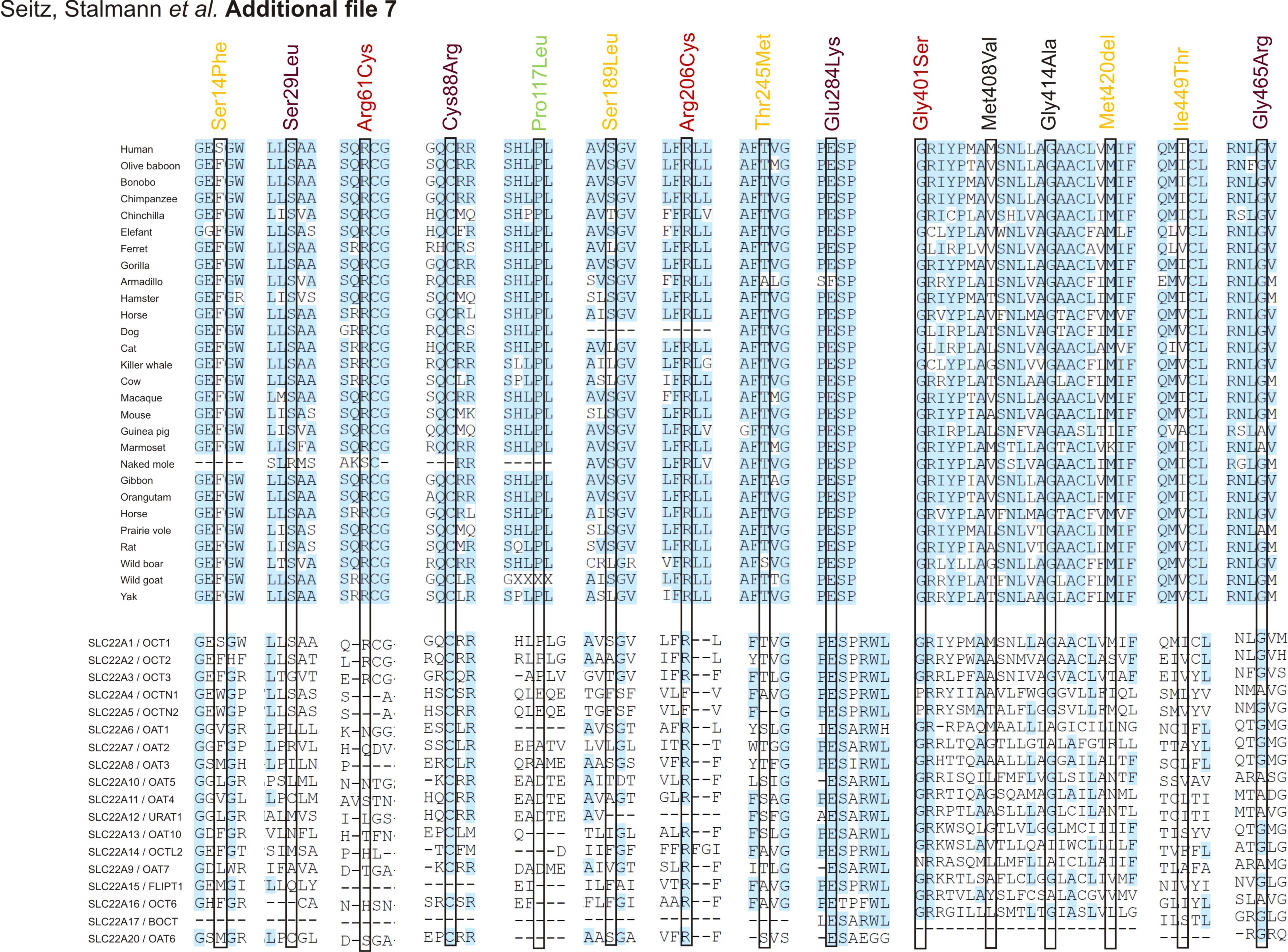

Supplement: Additional file 7: — Evolutionary conservation of the variable OCT1 sites in OCT1 orthologs (upper part) and paralogs (lower part). [file 13073_2015_172_MOESM7_ESM.jpg]

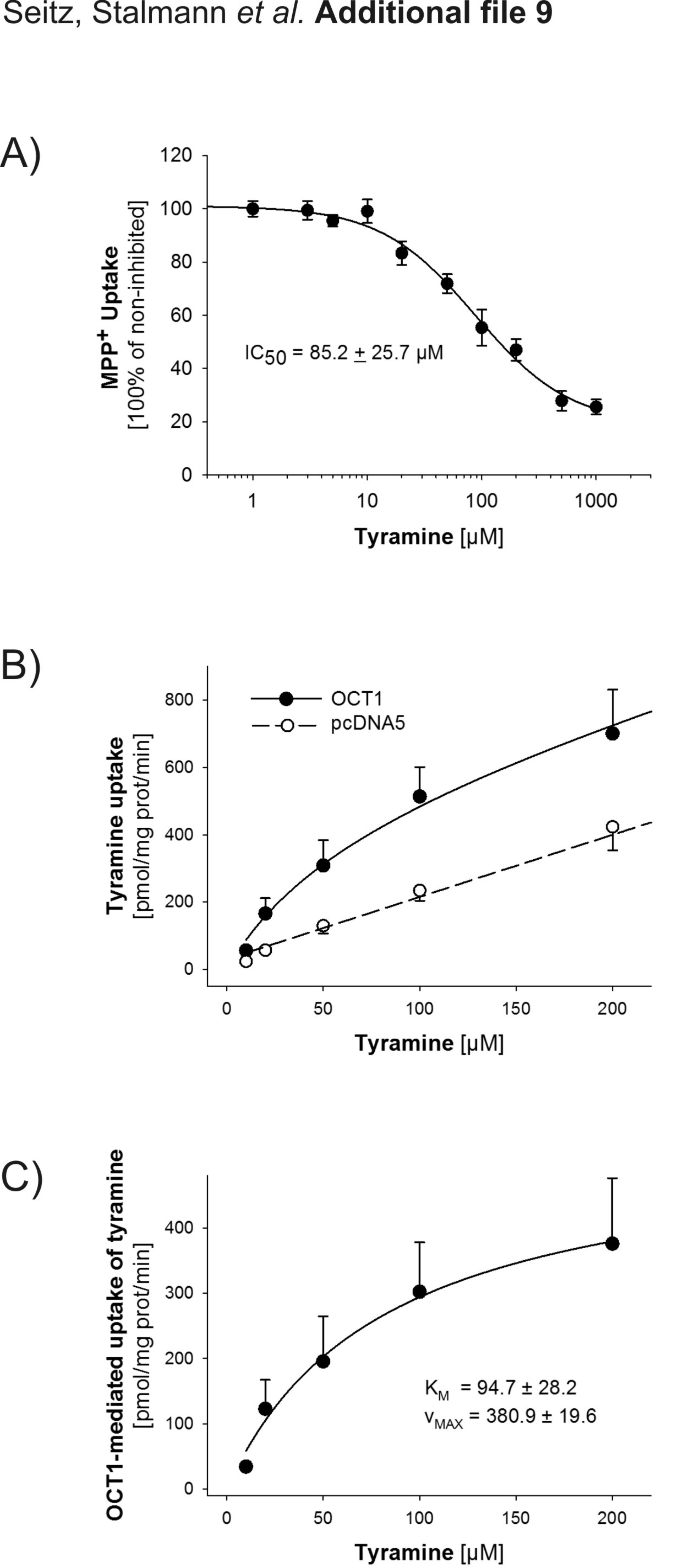

Supplement: Additional file 9: — Characterization of tyramine uptake in HEK 293 cells overexpressing OCT1. (a) Tyramine inhibits the uptake of MPP+. The cellular accumulation of 1-methyl-4-phenylpyridinium (MPP+) was measured after 1 min exposure to 10 nM 3H MPP+ in cells overexpressing OCT1*1. The graph shows mean values and standard errors of the mean from three independent experiments. (b) Concentration dependence of tyramine uptake. OCT1*1-overexpressing and control cells (stably transfected with the empty pcDNA5.1/FRT plasmid were incubated for 1 min with increasing concentrations of tyramine. The graphs show mean values and standard errors of the mean of three independent experiments. (c) The OCT1-mediated uptake. The OCT1-mediated fraction of the total uptake was calculated by subtracting the uptake in the control cells from the uptake in OCT1*1-overexpressing cells using the dataset shown in (b). [file 13073_2015_172_MOESM9_ESM.jpg]

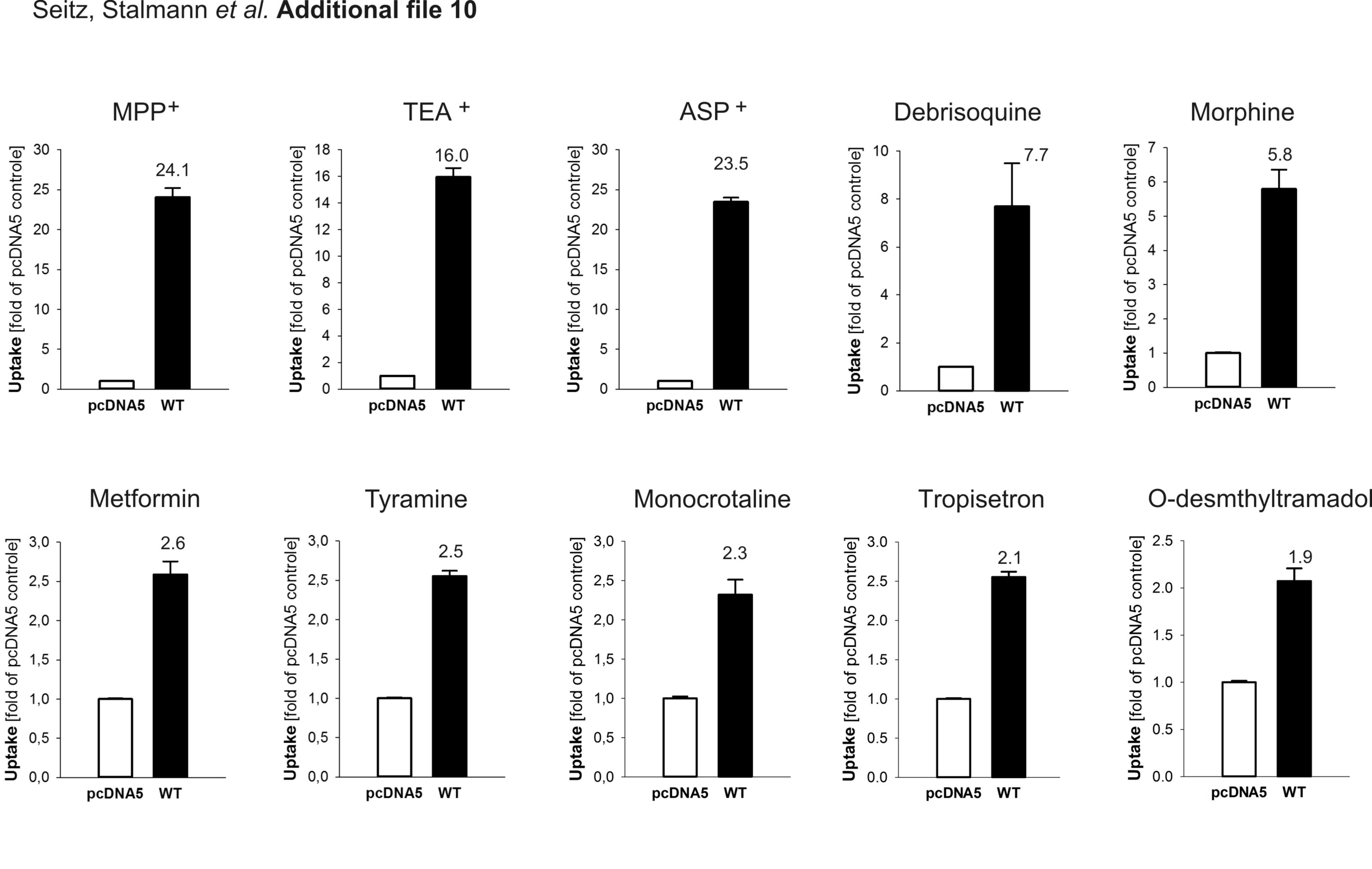

Supplement: Additional file 10: — Differences in the uptake between OCT1*1 overexpressing (WT) and control cells stably transfected with the empty pcDNA5.1/FRT plasmid (pcDNA5). The graphs show mean values and standard errors of the mean of three or more independent experiments. [file 13073_2015_172_MOESM10_ESM.jpg]

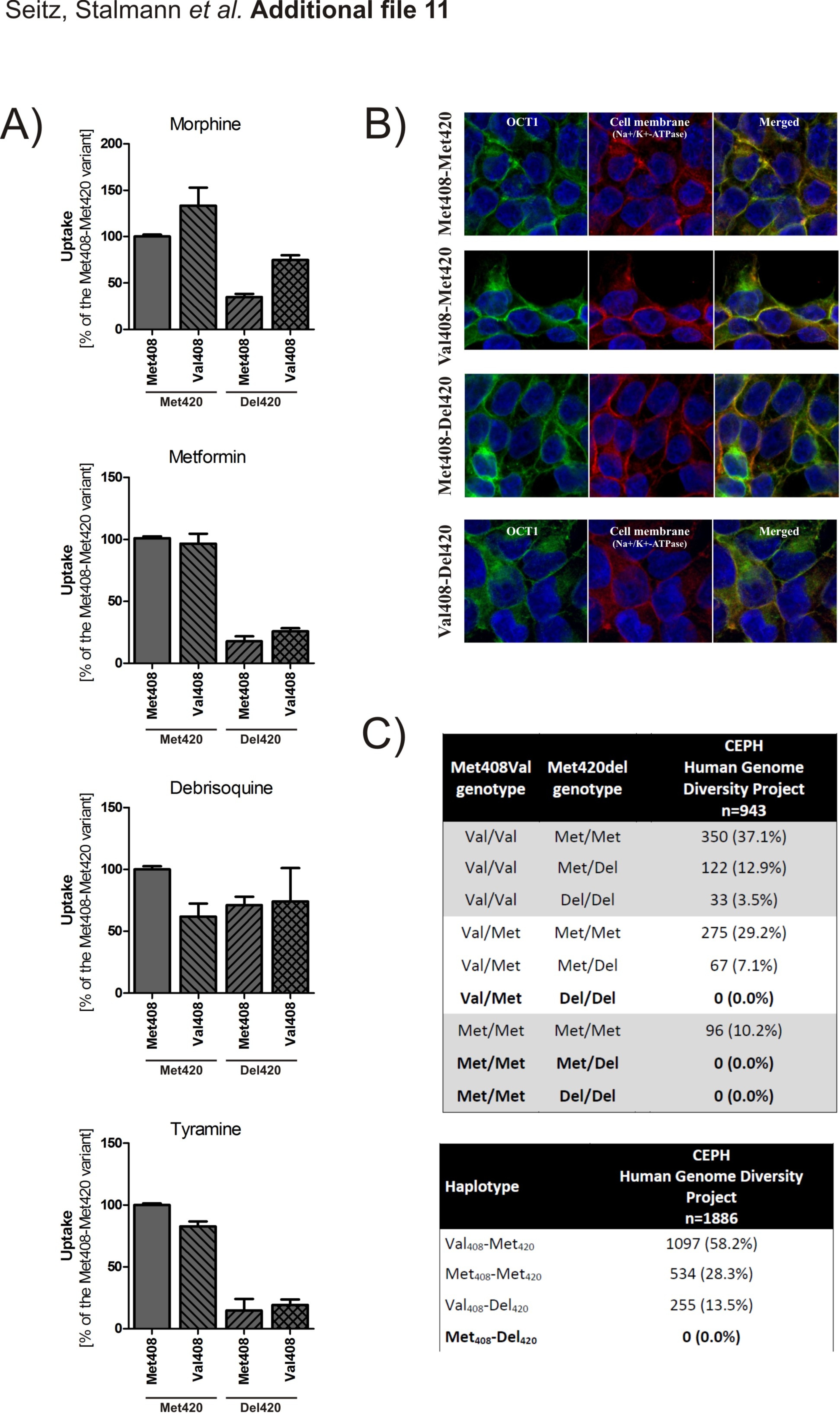

Supplement: Additional file 11: — Lack of interaction between Met408Val and Met420del polymorphisms in the OCT1 gene. (a) Comparison of the uptake activity of OCT1 carrying all theoretically possible combinations of the Met420del and Met408Val polymorphisms. The uptake was measured in HEK293 cells stably transfected with the OCT1 variants or with an empty control plasmid pcDNA5. Shown are means and standard error of the means of at least three independent experiments. (b) Subcellular localization of OCT1 isoforms carrying the four different combination of the Met420del and Met408Val polymorphisms. The protein is located on the plasma membrane indicated by co-staining with anti-Na+/K+ ATPase as a plasma membrane marker. No qualitative differences could be observed between cells carrying Met408 or Val408 independent from their background (Met420 or Del420). (c) Frequencies of the different single polymorphism genotypes (the upper part) and haplotype combinations (the lower part) in the HGDP-CEPH sample. Individual haplotypes were inferred using PHASE version 2.1. The genotype/haplotype frequencies are given as number (and %) of all individuals. [file 13073_2015_172_MOESM11_ESM.jpg]

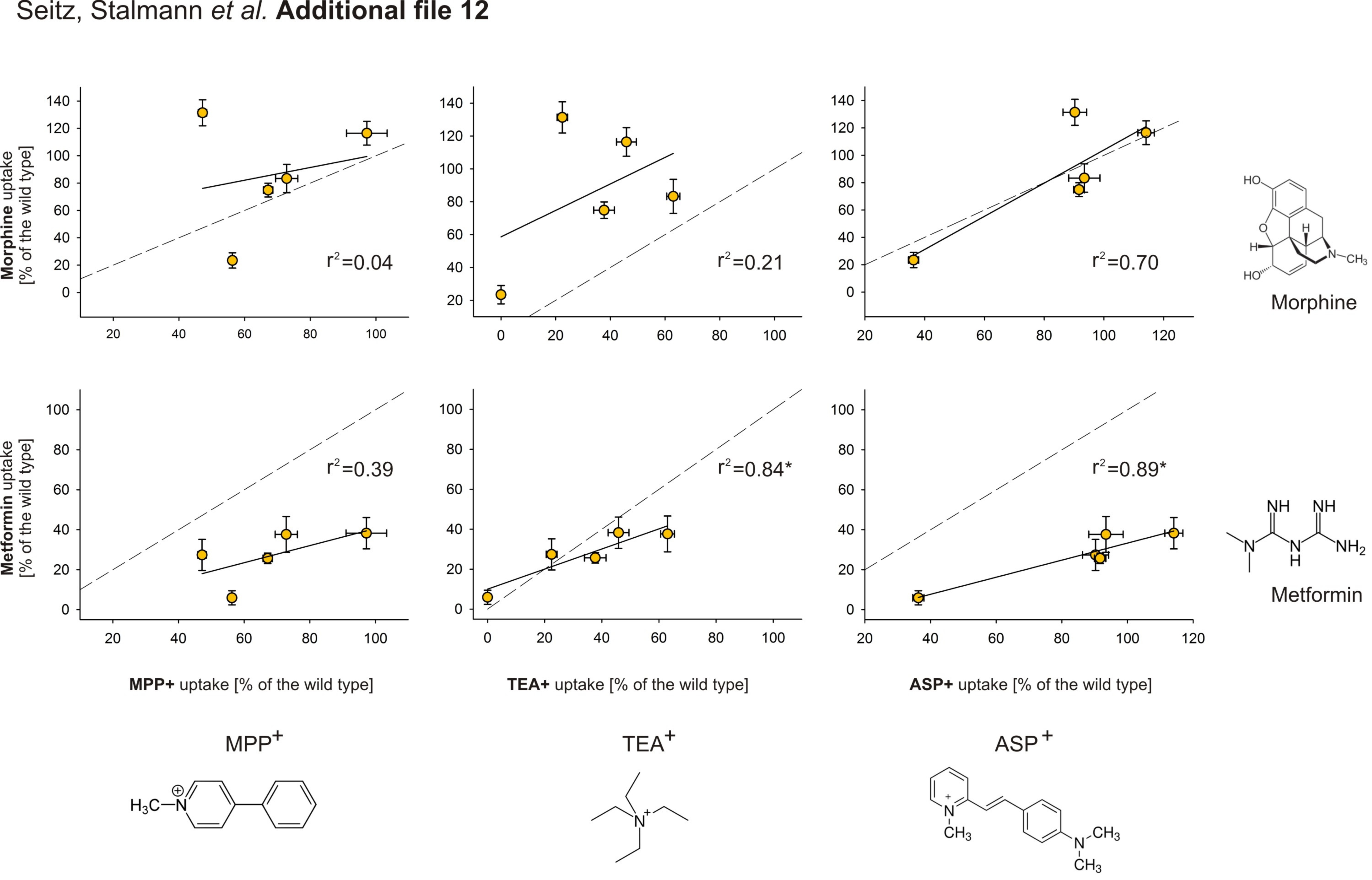

Supplement: Additional file 12: — Correlation between the effects of substrate-specific loss of function OCT1 alleles on the transport of model substrates and clinically relevant drugs. We analyzed correlations between the uptake of model OCT1 substrates (MPP+, TEA+, ASP+) and clinically relevant drugs (metformin and morphine) for all the OCT1 alleles that show substrate-specific loss of activity, that is, *2, *7, *10, *11, and *13. Shown are means and standard error of the means of at least three independent transport experiments. Solid lines represent linear regression, dashed lines represent an optimal theoretical correlation with identical effects of the loss-of-function allele on both the model substrates and drug. * denotes significant correlations with P <0.05. [file 13073_2015_172_MOESM12_ESM.jpg]

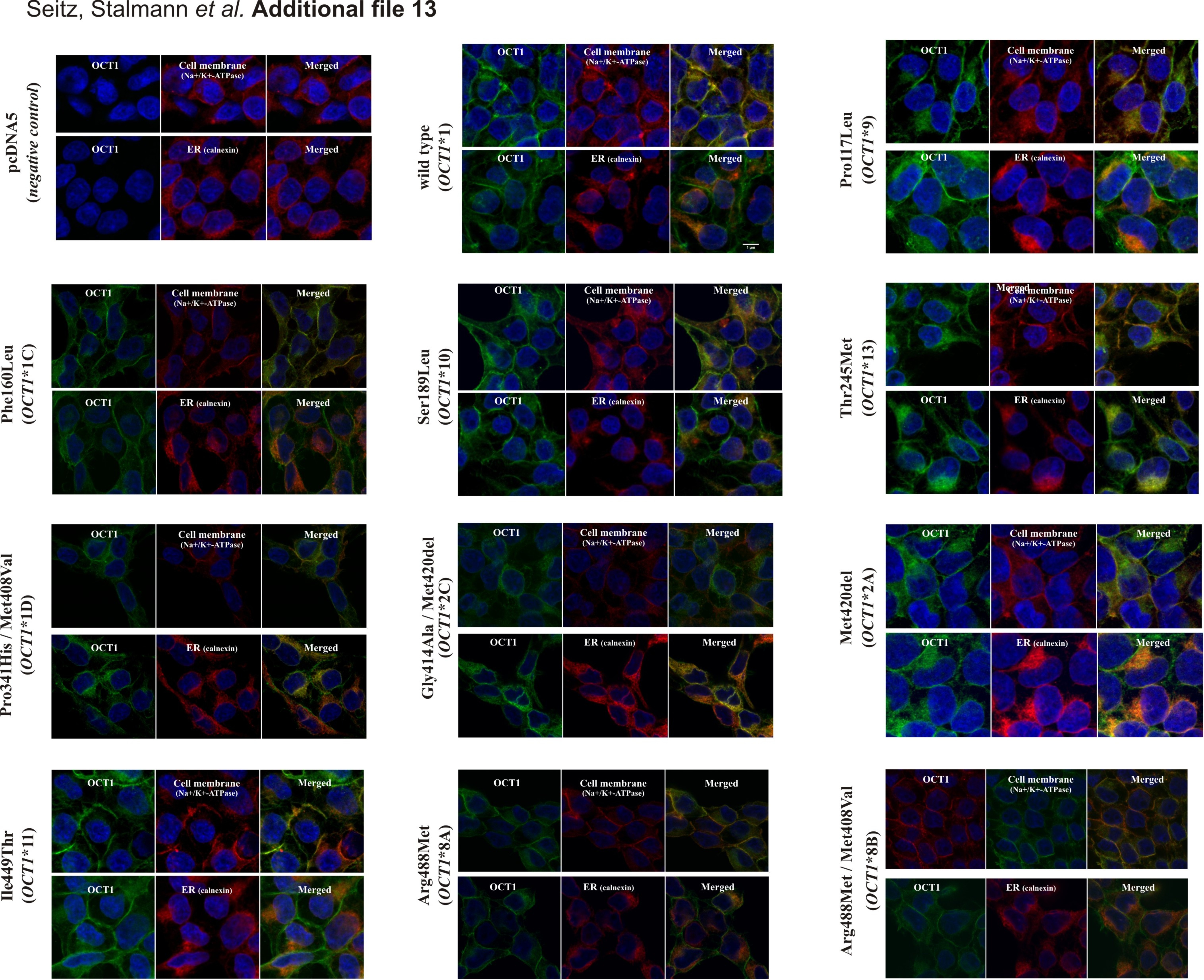

Supplement: Additional file 13: — Correct subcellular localization of OCT1 alleles *1, *2, *8, *9, *10, *11, and *13. The analyzed alleles are known to have normal (OCT1*1, *1B, *1C, and *1D), increased (OCT1*8 and *9) or substrate-specific loss of OCT1 activity (*2, *10, *11, and *13). The subcellular localization of the different OCT1 isoforms was analyzed after immunocytochemical staining of OCT1 (green) in combination with Na+/K+ ATPase (red, upper part) as a marker for plasma membrane and calnexin (red, lower part) as a marker for endoplasmic reticulum. [file 13073_2015_172_MOESM13_ESM.jpg]

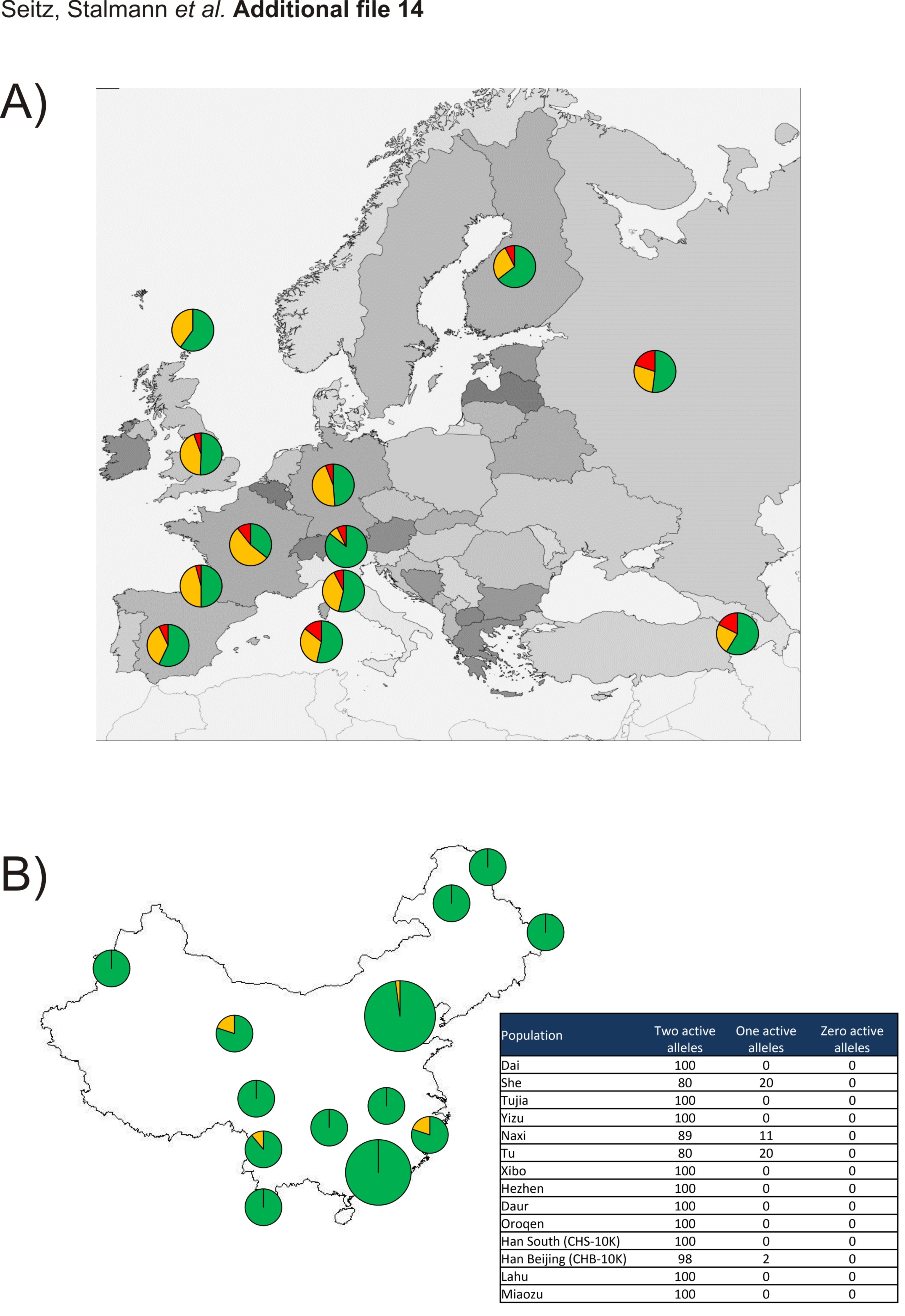

Supplement: Additional file 14: — Detailed maps of the interpopulation variation of the frequency of loss of OCT1 activity in Europe (a) and China (b). [file 13073_2015_172_MOESM14_ESM.jpg]

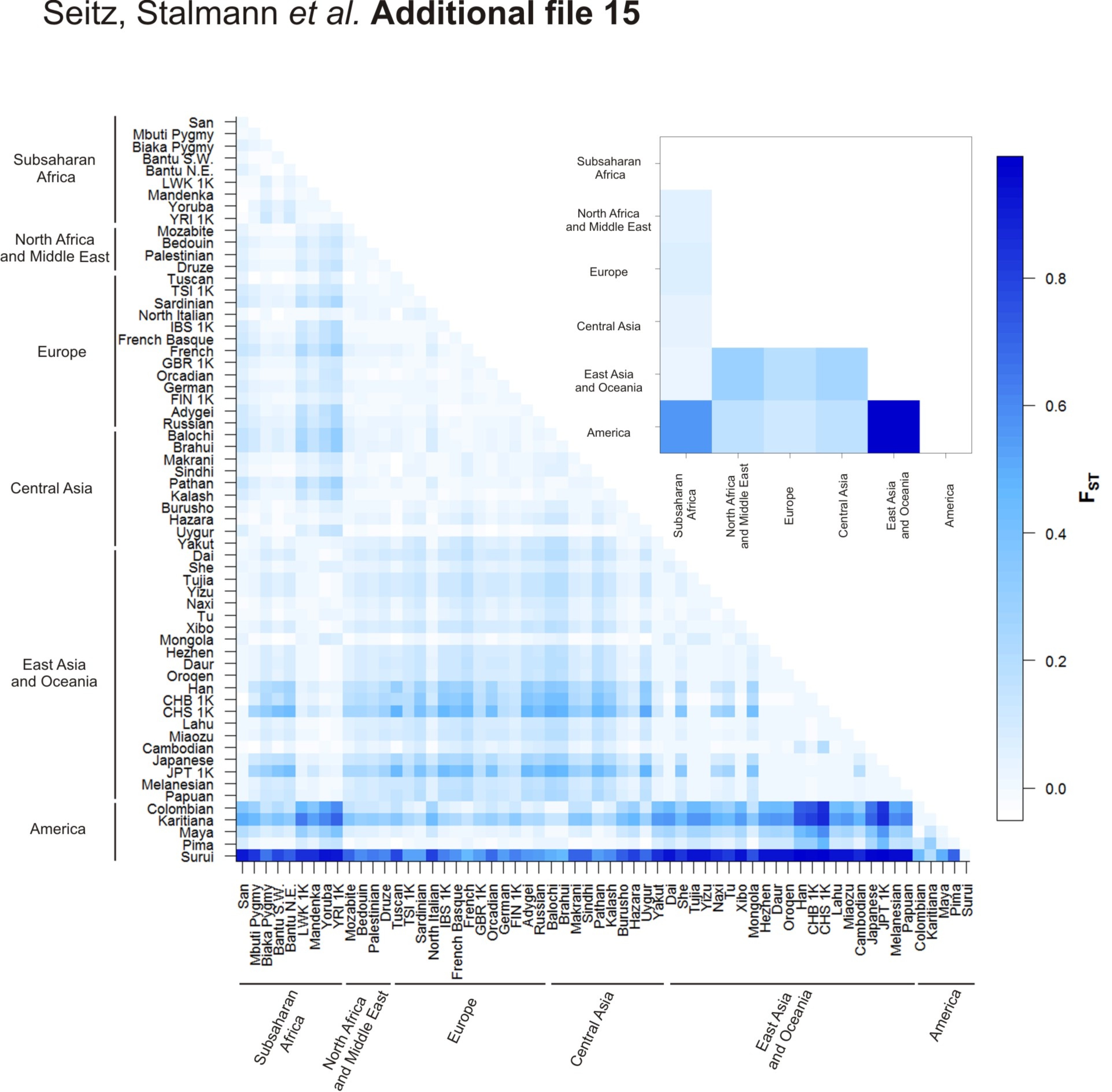

Supplement: Additional file 15: — Pairwise analyses of divergence based on the frequencies of OCT1 alleles in populations and in different world regions (the upper panel). The deep blue color correspond to high divergence between the populations / regions (high FST values). We analyzed 62 population from the 1000 Genomes Project (designated with 1 K) and the HGDP-CEPH project (the rest). The populations were stratified in world regions as listed in Table 1. In contrast to Fig. 7, which shows divergence at the level of loss of OCT1 activity, this figure shows divergences at the level of single OCT1 alleles. [file 13073_2015_172_MOESM15_ESM.jpg]
