# Supplementary material for: Global genetic analyses reveal strong inter-ethnic variability in the loss of activity of the organic cation transporter OCT1
Source: Genome Med. 2015 Jun 18;7(1):56. doi: 10.1186/s13073-015-0172-0 (PMC4495841; doi:10.1186/s13073-015-0172-0)
Supplement: Additional file 3: — List of the different tools used for prediction of the effects of OCT1 polymorphisms on protein function. [file 13073_2015_172_MOESM3_ESM.docx]

**Table S4: List of the different tools used for prediction of the effects of OCT1 polymorphisms on protein function**

| **Tool (Abbreviation)** | **Tool (Complete name)** | **Web site** | **Reference** | **Cut-off score for predicting substitution as deleterious ^*^** |
| --- | --- | --- | --- | --- |
| PROVEAN | Protein Variation Effect Analyzer | <http://provean.jcvi.org/genome_submit.php> | [1] | < 0.000 |
| SIFT | Sort Intolerant from Tolerant amino acid substitutions | <http://provean.jcvi.org/genome_submit.php> |  | < 0.050 |
| SNAP | Effects of single amino acid substitutions on protein function | <https://rostlab.org/services/snap/> | [2] |  |
| PolyPhen-2 | Polymorphisms Phenotyping v2 | <http://genetics.bwh.harvard.edu/pph2/bgi.shtml> | [3] |  |
| MutPred |  | <http://mutpred.mutdb.org/> | [4] | g > 0.500 |
| Mutation t@sting | MutationTaster2 | <http://www.mutationtaster.org/> | [5] |  |
| PhD SNP | Predictor of human Deleterious Single Nucleotide Polymorphisms | <http://snps.biofold.org/phd-snp/phd-snp.html> |  |  |
| SNPs3D |  | <http://www.snps3d.org/> | [6] | < 0.000 |

* The cut-off values are not given for the tools that predict directly as D (Deleterious) or n (Neutral).

References

1. Choi Y, Sims GE, Murphy S, Miller JR, Chan AP: **Predicting the functional effect of amino acid substitutions and indels.** *PLoS One* 2012, **7:**e46688.

2. Bromberg Y, Rost B: **SNAP: predict effect of non-synonymous polymorphisms on function.** *Nucleic Acids Res* 2007, **35:**3823-3835.

3. Adzhubei IA, Schmidt S, Peshkin L, Ramensky VE, Gerasimova A, Bork P, Kondrashov AS, Sunyaev SR: **A method and server for predicting damaging missense mutations.** *Nat Methods* 2010, **7:**248-249.

4. Li B, Krishnan VG, Mort ME, Xin F, Kamati KK, Cooper DN, Mooney SD, Radivojac P: **Automated inference of molecular mechanisms of disease from amino acid substitutions.** *Bioinformatics* 2009, **25:**2744-2750.

5. Schwarz JM, Rodelsperger C, Schuelke M, Seelow D: **MutationTaster evaluates disease-causing potential of sequence alterations.** *Nat Methods* 2010, **7:**575-576.

6. Yue P, Melamud E, Moult J: **SNPs3D: candidate gene and SNP selection for association studies.** *BMC Bioinformatics* 2006, **7:**166.
