# Supplementary material for: Global genetic analyses reveal strong inter-ethnic variability in the loss of activity of the organic cation transporter OCT1
Source: Genome Med. 2015 Jun 18;7(1):56. doi: 10.1186/s13073-015-0172-0 (PMC4495841; doi:10.1186/s13073-015-0172-0)
Supplement: Additional file 6: — In silico prediction of functional impact of amino acid substitutions observed in the worldwide genetic analyses of OCT1. [file 13073_2015_172_MOESM6_ESM.docx]

**Table S6. *In silico* prediction of functional impact of amino acid substitutions observed in the worldwide genetic analyses of OCT1.**

| **Amino acid substitution** | **Position on chr. 6** | **dbSNP ID** | **PROVEAN** | **SIFT** | **SNAP** | **PPH2** | **MutPred** | **SNPs3D** | **Mutation t@sting** | **PhD SNP** | **Number of deleterious predictions** |
| --- | --- | --- | --- | --- | --- | --- | --- | --- | --- | --- | --- |
| **Ser14Phe** | 160543008 | rs34447885 | n | n | n | n | D | n | n | n | 1/8 |
| **Leu23Val** | 160543034 | rs34570655 | n | n | n | n | D | n | - | n | 1/7 |
| **Ser29Leu** | 160543053 | rs375175439 | D | D | n | D | D | n | D | n | 5/8 |
| **Arg61Cys** | 160543148 | rs12208357 | D | D | D | D | D | D | n | D | 7/8 |
| **Ala80Val** | 160543206 | rs145740120 | n | n | n | n | n | n | n | n | 0/8 |
| **Cys88Arg** | 160543229 | rs55918055 | D | D | D | D | D | D | D | D | 8/8 |
| **Pro117Leu** | 160543317 | *rs200684404* | D | D | D | D | D | n | D | n | 6/8 |
| **Leu160Phe** | 160551204 | rs683369 | n | n | n | n | D | n | n | D | 2/8 |
| **Phe161Leu** | 160551205 | *novel* | n | n | n | n | n | n | n | D | 1/8 |
| **Gly165Cys** | 160551217 | rs201942835 | n | n | n | n | n | n | n | D | 1/8 |
| **Ala187Val** | 160553308 | rs374227992 | n | n | n | n | D | n | n | n | 1/8 |
| **Ser189Leu** | 160553314 | rs34104736 | D | n | n | n | D | n | n | D | 3/8 |
| **Arg206Cys** | 160553364 | *not available** | D | D | D | D | D | D | D | D | 8/8 |
| **Gly220Val** | 160553407 | rs36103319 | D | D | D | D | D | n | D | D | 7/8 |
| **Thr245Met** | 160555052 | *novel* | D | D | n | D | D | D | n | D | 6/8 |
| **Phe273Leu** | 160555137 | *novel* | D | n | n | n | D | n | D | n | 3/8 |
| **Glu284Lys** | 160557262 | *novel* | D | D | D | D | D | D | D | D | 8/8 |
| **Pro341Leu** | 160557643 | rs2282143 | D | D | D | D | D | D | D | D | 8/8 |
| **Arg342His** | 160557646 | rs34205214 | n | D | n | n | D | n | - | n | 2/7 |
| **Gly401Ser** | 160560824 | rs34130495 | D | D | D | D | D | D | D | D | 8/8 |
| **Ile403Val** | 160560830 | rs188898744 | n | n | n | n | D | D | n | n | 2/8 |
| **Met408Val** | 160560845 | rs628031 | n | n | n | n | D | n | n | n | 1/8 |
| **Gly414Ala** | 160560864 | rs72552762 | D | D | n | D | D | D | D | D | 7/8 |
| **Val419Ile** | 160560878 | rs200915918 | n | n | n | n | n | n | n | n | 0/8 |
| **Ile449Thr** | 160564642 | rs183240019 | D | D | D | D | D | D | n | D | 7/8 |
| **Val461Ile** | 160564677 | rs34295611 | n | n | n | n | D | n | n | n | 1/8 |
| **Gly465Arg** | 160575837 | rs34059508 | D | D | D | D | D | D | D | D | 8/8 |
| **Arg488Met** | 160575907 | rs35270274 | n | n | D | n | D | n | - | D | 3/7 |
| **Val519Phe** | 160577063 | rs78899680 | n | n | n | D | n | D | n | D | 3/8 |

* The variant Arg206Cys has been reported before, but has no dbSNP ID number (status of the dbSNP data base 08 March 2015)

D, deleterious; n, neutral
